# Brain network topology and neural mechanisms in Sanda athletes: a comparative study across training levels

**DOI:** 10.3389/fnins.2026.1801936

**Published:** 2026-09-04

**Authors:** Yang Chen, Zifeng Jia, Zhangzhi Zhao, Jihe Zhou, Yuanzheng Lin, Bin Zhao

**Affiliations:** 1 Chengdu Sport University, Chengdu, China; 2 University of Electronic Science and Technology of China, Chengdu, China

**Keywords:** brain networks, graph theory, neural mechanisms, performance level, Sanda athletes

## Abstract

**Purpose:**

This study investigated the topological organization of brain networks in Sanda athletes across different training levels to elucidate the neural mechanisms underlying the sport’s exceptional sensorimotor integration and training-related neural plasticity.

**Methods:**

Multimodal magnetic resonance imaging (MRI) data were collected from 35 participants, including elite athletes, second-level athletes, and novices. Using the Automated Anatomical Labeling 116-region atlas (AAL116), structural connectivity (SC) networks were constructed based on fractional anisotropy (FA) derived from diffusion tensor imaging, while functional connectivity (FC) networks were computed using Pearson correlations between regional blood-oxygen-level-dependent (BOLD) time series. Structure–function coupling (SFC) networks were generated as a weighted linear combination of SC and FC. Network differences were assessed using Network-Based Statistic (NBS) and graph-theoretical analyses.

**Results:**

Elite athletes exhibited prominent structure–function coupling within visual–motor–executive pathways, whereas novices showed greater involvement of language–rhythm-related pathways. At the global level, elite athletes demonstrated stronger small-world organization and network synchrony, suggesting more efficient information transmission. At the nodal level, the parietal–cerebellar system emerged as a critical hub across the examined networks, with particularly prominent involvement of the right supramarginal gyrus (SMG.R) and right cerebellar lobule VI (CRBL6.R).

**Discussion:**

These regions are likely to play a central role in sensorimotor integration and rhythm control. These findings confirm that long-term Sanda training optimizes brain network topology, providing key insights into the neural plasticity of athletic expertise.

## 1 Introduction

Growing evidence indicates that long-term physical training can induce substantial neuroplastic changes in both brain structure and function. These adaptations are not limited to localized brain regions but extend to the reorganization of large-scale brain networks, thereby supporting motor performance and higher-order cognitive processes (Kolb and Whishaw, 1998; Fuchs and Fluegge, 2014; Petersen and Sporns, 2015).

Importantly, different types of sports impose distinct cognitive and sensorimotor demands, which may lead to sport-specific patterns of neural adaptation. A fundamental distinction in sports science is between open-skill and closed-skill sports. Open-skill sports, such as combat sports and ball games, are typically performed in dynamic and unpredictable environments, requiring rapid decision-making, response inhibition, and continuous sensorimotor integration. In contrast, closed-skill sports, such as shooting, gymnastics, diving, and swimming, are usually performed in stable and predictable environments, with relatively fixed movement patterns and a greater reliance on internal proprioceptive feedback. Consequently, they generally place lower demands on executive control and perceptual flexibility (Zhou and Wang, 2024; Wang Z. et al., 2025).

As a representative open-skill combat sport, Sanda is characterized by high-intensity confrontation, rapid tactical switching, and complex perception-action coupling. Athletes must integrate visual, proprioceptive, and vestibular information within milliseconds while maintaining motor coordination and emotional regulation under competitive pressure (Zhao and Guan, 2014; Liu et al., 2025). These characteristics suggest that long-term Sanda training may induce specialized adaptations in large-scale brain networks, particularly those involved in sensorimotor processing, attentional control, and executive function.

From the perspective of network neuroscience, the human brain operates as a complex system that depends on a dynamic balance between functional segregation and functional integration (Petersen and Sporns, 2015; Wendelken et al., 2016). Large-scale networks, including the sensorimotor network (SMN), dorsal attention network (DAN), salience network (SN), and default mode network (DMN), interact dynamically to support goal-directed behavior (Smith et al., 2009). Training-induced plasticity is reflected not only in changes within localized brain regions but also in the reorganization of cross-network connectivity patterns, including structural connectivity (SC) and functional connectivity (FC) (Marek et al., 2015).

To further understand how anatomical structures support functional dynamics, structure–function coupling (SFC) has emerged as an important framework in recent neuroscience research. Compared with single-modality approaches, macroscale SFC provides a more comprehensive characterization of brain organization by revealing how structural constraints shape functional interactions across regions (Fotiadis et al., 2024). Moreover, recent studies have suggested that SFC exhibits substantial regional heterogeneity and dynamic variability, offering important insights into experience-dependent neural reorganization (Liu et al., 2022).

In the field of sports neuroscience, athletes provide an ideal model for investigating training-induced brain plasticity. Previous studies have shown that long-term training can lead to structural and functional adaptations in motor, visual, cerebellar, and prefrontal regions (Tan et al., 2017; Bai et al., 2020; Zhang et al., 2021; Yan et al., 2024). These changes are often associated with enhanced network integration, improved information transmission efficiency, and strengthened sensorimotor hubs, reflecting the neural basis of athletic expertise. In particular, neuroimaging studies of open-skill and fast-response sports have reported training-related alterations in frontoparietal, executive-control, attentional, and sensorimotor-related networks. These network-level adaptations may contribute to visuospatial processing, hand-eye coordination, perception-action coupling, and rapid decision-making in dynamic environments (Di et al., 2012; Tan et al., 2017; Gao et al., 2019; Yang et al., 2020).

Beyond general large-scale network organization, training-related neuroplasticity in open-skill combat sports may be expressed through several functionally relevant neurobiological systems. Neuroimaging evidence from athlete and open-skill training contexts indicates that parietal-cerebellar pathways are involved in multisensory integration, movement calibration, and motor coordination, which are relevant to the rapid updating of somatosensory, proprioceptive, and motor signals during action execution (Park et al., 2015; Tan et al., 2017; Zhang et al., 2021). Related evidence also suggests that frontoparietal control systems contribute to attentional reallocation, inhibitory control, and tactical adjustment under time pressure, functions that are behaviorally central to Sanda exchanges (Marek et al., 2015; Wendelken et al., 2016; Yan et al., 2024). In parallel, findings from athlete cohorts suggest that limbic-basal ganglia-cerebellar circuits may be associated with action selection, affective stability, and experience-dependent motor memory, providing a plausible neural basis for decision-execution coupling in combat performance (Gong et al., 2019; Zhang et al., 2021; Yan et al., 2024). Finally, because Sanda performance requires rapid transformation of external visual information into adaptive motor responses, visual-sensorimotor integration pathways may also play a central role (de Quel and Bennett, 2019; Russo and Ottoboni, 2019).

Despite these advances, the neural mechanisms underlying open-skill combat sports such as Sanda remain insufficiently understood. Existing studies have largely focused on single-modality analyses or non-combat sports, limiting our understanding of how different levels of brain networks jointly contribute to athletic performance. In particular, there remains a lack of studies integrating SC, FC, and SFC to systematically examine training-related brain network reorganization using network-based statistic (NBS) and graph-theoretical analysis.

To address this gap, the present study investigated the organization of brain networks in Sanda athletes across different performance levels. Based on multimodal magnetic resonance imaging (MRI) data, SC, FC, and SFC networks were constructed using the AAL116 brain atlas, and intergroup comparisons were conducted using NBS and graph-theoretical metrics. This approach was designed to clarify how long-term specialized training shapes brain network topology and to provide new evidence for understanding the neural mechanisms underlying Sanda expertise.

Based on this theoretical framework, we hypothesized that athletes with different training levels would show more pronounced network differences within specific neurobiological systems rather than a nonspecific whole-brain distribution. More specifically, we predicted that SC, FC, and SFC would show convergent alterations within the parietal–cerebellar sensorimotor loop, frontoparietal executive control network, limbic-basal ganglia-cerebellar circuit, and visual-sensorimotor integration network. Furthermore, we expected that elite athletes would demonstrate more optimized global integration and nodal hub properties, reflected by enhanced small-world organization, synchrony, centrality, and nodal efficiency, particularly in regions supporting perception-action coupling, executive control, and cerebellar-parietal integration.

## 2 Research participants and methods

### 2.1 Research participants

Participants were recruited from the Sports and Brain Science Experimental Platform of Chengdu Sport University, including multimodal MRI data, comprising T1-weighted images (T1w), resting-state functional magnetic resonance imaging (rs-fMRI), and diffusion tensor imaging (DTI).

A total of 35 healthy male participants (aged 17–29 years) were included and categorized into three groups based on training background and competitive level: elite athletes (*n* = 12), second-level athletes (*n* = 11), and a control group (*n* = 12) (Table 1). To minimize potential confounding effects, general physical activity levels were considered to ensure comparability across groups. The elite group consisted of athletes from the Sichuan Provincial Sanda Team, including 8 National Master Sportsmen and 4 National First-Class athletes. The second-level group consisted of National Second-Class athletes from the Chengdu Sport University Sanda Team, all with systematic training experience. The control group comprised undergraduate students (non-sports majors) with no history of martial arts or combat sports training and no engagement in systematic athletic training. All participants were healthy adult males with no history of neurological or psychiatric disorders and provided written informed consent. The study was approved by the Ethics Committee of Chengdu Sport University [Approval No.: (2025)10].

**Table 1:** Demographic characteristics of participants.

Group	Number	Age
Elite athletes	12	20.5 ± 3.4
Second-level athletes	11	19.8 ± 2.9
Ordinary college students	12	19.2 ± 0.8

### 2.2 MRI data acquisition

All imaging data were acquired using a 3.0 T Philips Ingenia Elition X superconducting magnetic resonance imaging system. During the scanning, participants lay supine in the MRI scanner with their eyes closed, remaining awake and as still as possible to minimize cognitive engagement and motion-related artifacts. The acquisition sequences and parameters were as follows:

Structural images, T1W-3D-TFE: repetition time (TR) = 6.4 ms; echo time (TE) = 2.8 ms; Flip angle: 8°; field of view (FOV) = 256 × 256 × 175 mm^3^; matrix size: 256 × 256; spatial resolution: 1.0 × 1.0 × 1.0 mm^3^; slice thickness: 1.0 mm; no gap; 175 slices.

rs-fMRI data were acquired using a blood-oxygen-level-dependent (BOLD) contrast echo-planar imaging (EPI) sequence: TR = 1988 ms; TE = 35 ms; flip angle: 90°; 50 slices; slice thickness: 3.2 mm; no gap; FOV = 240 × 240 × 160 mm^3^; matrix size: 64 × 62; Voxel size: 3.75 × 3.89 × 3.2 mm^3^; 240 time points were acquired.

DTI was performed using an EPI sequence: TR = 5,134 ms; TE = 97 ms; FOV = 224 × 224 × 170 mm^3^; matrix size: 112 × 110; Voxel size: 2.0 × 2.04 × 2.0 mm^3^; 85 slices; no gap; diffusion sensitivity coefficient (*b*-value) = 1,000 s/mm^2^, 32 diffusion directions, acquisition of b = 0 images.

### 2.3 Data preprocessing pipeline

The data used in this study were processed using fMRIPrep for structural and functional image data. fMRIPrep is a software package within the NiPreps project, designed for preprocessing task-based and resting-state fMRI data (Esteban et al., 2019). Functional MRI data were preprocessed using fMRIPrep, a robust and standardized pipeline that integrates multiple neuroimaging software packages, including FSL, Advanced Normalization Tools (ANTs), FreeSurfer, and Analysis of Functional NeuroImages (AFNI).

Specifically, both structural and functional images were nonlinearly normalized to the MNI152NLin2009cAsym standard space using ANTs. To control for head motion effects, outlier volumes were identified based on framewise displacement (FD) and standardized DVARS. Volumes with FD > 0.5 mm or standardized DVARS > 1.5 were defined as motion outliers and subsequently included as nuisance regressors in further analyses.

Slice timing correction was automatically performed based on acquisition parameters provided in the Brain Imaging Data Structure (BIDS) metadata. Given that regional time series were extracted using the AAL116 atlas, no spatial smoothing was applied prior to network construction to avoid potential signal contamination across adjacent brain regions.

For nuisance regression, anatomical CompCor (aCompCor) was employed. Principal components explaining 50% of the variance from white matter (WM) and cerebrospinal fluid (CSF) signals were regressed out, along with 24 head motion parameters (including the six rigid-body motion parameters, their temporal derivatives, and quadratic terms), to further reduce motion-related and physiological noise.

Diffusion MRI data preprocessing and structural connectivity network construction were performed using a combination of FMRIB Software Library (FSL) and DSI Studio (Jenkinson et al., 2012; Yeh et al., 2013). First, eddy current distortions and head motion were corrected using the eddy_correct tool in FSL, by registering all diffusion-weighted images to the b0 image via affine transformation. Subsequently, brain extraction was conducted using bet2 (fractional intensity threshold = 0.35) to generate a brain mask, followed by tensor fitting using dtifit to obtain diffusion metrics such as fractional anisotropy (FA).

Based on the preprocessed data, whole-brain white matter fiber reconstruction and structural connectivity network construction were performed using DSI Studio. A deterministic fiber tracking algorithm based on the DTI model was applied. This approach was selected due to its high tracking stability and computational efficiency under high signal-to-noise conditions, making it suitable for group-level structural connectivity analysis.

The fiber tracking parameters were set as follows: step size = 1 mm, turning angle = 60°, smoothing = 1, and fiber length range = 30–300 mm. Fiber tracking was terminated after generating 40,000 streamlines. Using the AAL116 atlas, each brain region was defined as a network node. The SC matrix was constructed at the individual level, with the number of fibers or the mean FA value between each pair of regions used as the edge weight, and subsequently used for network analysis.

### 2.4 Research method

**Figure 1:**
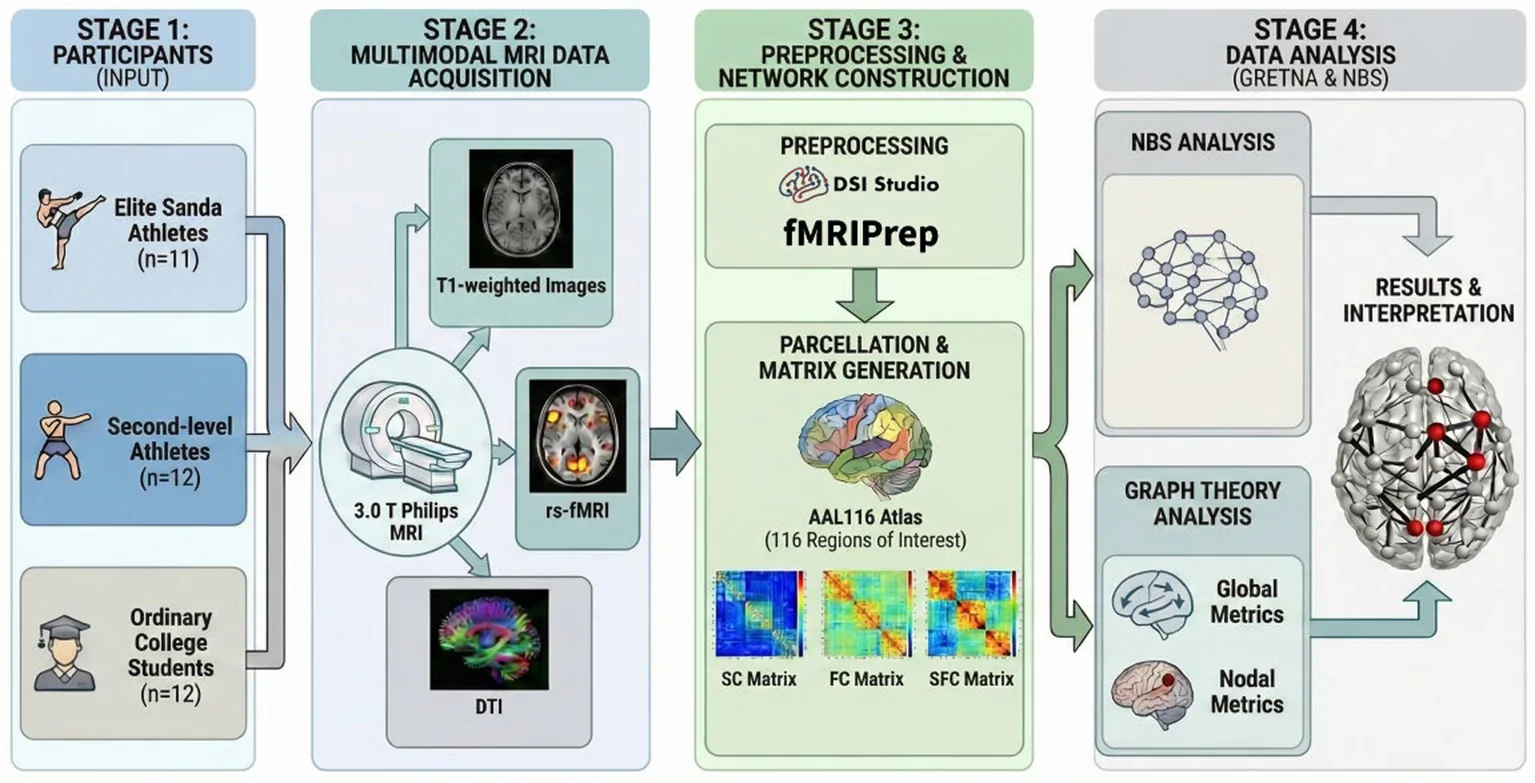
The schematic flowchart of the study design. The pipeline integrates participant selection, multimodal MRI acquisition, preprocessing, network construction, and dual-branch analysis using NBS and graph theory.

The overall research framework and data processing pipeline are illustrated in Figure 1. The study followed a systematic workflow consisting of four stages: (1) Participant Grouping, where participants were categorized into elite athletes, second-level athletes, and ordinary students; (2) Data Acquisition, involving the collection of multimodal MRI data (T1, rs-fMRI, DTI); (3) Preprocessing and Network Construction, where data were processed using fMRIPrep and DSI Studio to construct SC, FC, and SFC matrices based on the AAL116 atlas; and (4) Statistical & Topological Analysis, utilizing NBS for subnetwork identification and Graph Theoretical Network Analysis (GRETNA) for global and nodal graph theory metric calculations.

#### 2.4.1 Construction of connectivity matrices based on brain atlases

Before analysis, a functional connectivity matrix based on the atlas needs to be constructed. According to the preprocessing procedure, this study uses functional and diffusion imaging data to calculate the functional connectivity matrix and structural connectivity matrix in voxel space, as well as the SFC matrix obtained by coupling the FC and SC matrices. The FC matrix is calculated based on the preprocessed fMRI data by extracting the BOLD time series from three-dimensional voxels and calculating the Pearson correlation coefficient between them to construct the functional connectivity matrix. The SC matrix is based on the preprocessed diffusion magnetic resonance imaging data, using fiber tracking methods to reconstruct the white matter fiber bundle network, and using mean FA between connected voxels as the connection strength indicator to construct the structural connectivity matrix.

Functional connectivity is defined based on the Pearson correlation coefficient between the signals of two ROIs. For subsequent network construction and graph-theoretical analysis, only positive correlations were retained. Specifically, negative FC values were set to zero before thresholding. The calculation method of the Pearson correlation coefficient is shown in Equation 1:


$${\dot{c}}_{\mathrm{ij}}=\frac{\mathrm{cov}(S_{i},S_{j})}{}$$ (1)

Where $S_{i}$ and $S_{j}$ represent regions i and j , cov(⋅) represents the covariance, and $\sigma_{S_{i}}$ and $\sigma_{S_{j}}$ represent the standard deviations of the signals.

FA is an important parameter obtained from DTI scans. It is defined as the ratio of the anisotropic component of the diffusion tensor to the entire diffusion tensor, and its calculation method is shown in Equation 2:


$$\mathrm{FA}=\sqrt{\frac{{\left(\lambda_{1}-\lambda_{2}\right)}^{2}+{\left(\lambda_{2}-\lambda_{3}\right)}^{2}+{\left(\lambda_{1}-\lambda_{3}\right)}^{2}}{2(\lambda_{1}^{2}+\lambda_{2}^{2}+\lambda_{3}^{2})}}$$ (2)

Where $\lambda_{1}$ , $\lambda_{2}$ , and $\lambda_{3}$ are the three eigenvalues of the diffusion tensor, corresponding to the diffusion intensity in the main diffusion direction and two perpendicular directions.

The construction of SFC involves a weighted sum of SC and FC, and its calculation method is shown in Equation 3:


SFC=α⋅FC+(1−α)⋅SC (3)

The value of α is in the range [0,1] , representing the weight during SFC construction. It should be noted that the term SFC has been defined in different ways across the literature. In many previous studies, SFC refers to the correspondence or association between structural and functional connectivity patterns (Fuchs and Fluegge, 2014). In the present study, SFC is operationally defined as a weighted integration of SC and FC matrices to characterize their combined network organization. To evaluate the influence of the weighting coefficient, we performed a sensitivity analysis across α = 0.1–0.9, and the results are provided in Supplementary Figure S1 and Supplementary Tables S1,S2. Relative to alpha = 0.3, the pairwise t-statistic patterns showed moderate-to-high similarity across the tested range (*r* = 0.652–1.000), with the highest consistency observed near alpha = 0.2–0.4. However, the number and identity of supra-threshold edges varied across alpha values (1–6 edges across comparisons), and edge-set overlap decreased at larger alpha values. Therefore, the SFC findings should be interpreted as alpha-dependent multimodal coordination patterns rather than parameter-free biological effects. The main conclusion is that group differences are not restricted to a single isolated alpha value, but the precise edge-level topology should be interpreted with appropriate sensitivity to the chosen weighting coefficient.

#### 2.4.2 Network connectivity analysis based on NBS

In this study, the NBS approach was applied to the SC, FC, and SFC matrices to identify significant subnetworks exhibiting group differences. Instead of testing each connection in isolation, NBS controls the Family-Wise Error Rate (FWER) at the network level, effectively addressing the multiple comparisons problem in high-dimensional connectome data. Specifically, pairwise statistical comparisons among the three groups were directly conducted using independent two-sample t-tests within the General Linear Model (GLM) framework. For each pairwise comparison, a primary uncorrected connection-level threshold of *p* < 0.001 was applied to the connectivity matrices to define a set of suprathreshold links and identify connected components. Subsequently, to rigorously control for multiple comparisons, non-parametric permutation testing with 5,000 permutations was performed on these components to estimate their empirical null distribution. Finally, subnetworks were considered statistically significant if their FWER-corrected *p*-value was less than 0.05.

#### 2.4.3 Graph theory metric analysis based on GRETNA

Graph theory attributes of the resting-state FC, SC, and SFC networks were systematically evaluated using the GRETNA toolbox (Wang et al., 2015). To eliminate the potential bias introduced by selecting a single arbitrary threshold, a continuous range of sparsity thresholds was applied to the individual connectivity matrices. As defined previously, the sparsity thresholds ranged from 0.05 to 0.50 with a step size of 0.01.

At each sparsity threshold, we extracted global network metrics including small-worldness, global efficiency, local efficiency, assortativity, synchronizability, and hierarchy, together with nodal network metrics. Detailed definitions and mathematical formulas for these global and nodal metrics are summarized in Tables 2, 3, respectively.

**Table 2:** Common global network metrics.

Global network metrics	Meaning	Mathematical definition
Small-worldness	A type of network architecture that simultaneously exhibits high local clustering and short path length.	$\alpha =\frac{C/C_{\mathrm{rand}}}{L/L_{\mathrm{rand}}}$ Where C and L are the clustering coefficient and characteristic path length of the network, and $C_{\mathrm{rand}}$ and $L_{\mathrm{rand}}$ are the mean values of matched random networks.
Global efficiency	Reflects the efficiency of parallel information transfer across the entire network.	$E_{\text{glob}}=\frac{1}{N(N-1)}\sum_{i\neq j\in G}\frac{1}{d_{\mathrm{ij}}}$
Local efficiency	Reflects the efficiency of information transfer within local neighborhoods of the network.	$E_{\mathrm{loc}}(G)=\frac{1}{N}\sum_{i=1}^{N}E_{\text{glob}}(G_{i})$ where $G_{i}$ is the subgraph formed by the neighbors of node i.
Assortativity	Indicates the tendency of nodes with similar degrees to connect preferentially to one another.	r=Pearson correlation coefficient of the degrees of all connected node pairs.
Synchronizability	Measures the degree of coordination among node activities in the network.	$S=\frac{\lambda_{2}}{\lambda_{N}}$ Where $\lambda_{2}$ and $\lambda_{N}$ are the second smallest and the largest eigenvalues of the network’s Laplacian matrix.
Hierarchy	An important metric used to identify whether the network possesses a hierarchical, layered organizational structure.	β Derived from the power-law relationship between the clustering coefficient $C_{i}$ and degree $k_{i}$ : $C\approx k^{-\beta }$ .

**Table 3:** Common nodal network metrics.

Nodal network metrics	Meaning	Mathematical definition
Clustering coefficient	Measures the extent to which the neighbors of a given node are interconnected, reflecting the likelihood of forming triangular connections.	$C_{i}=\frac{2t_{i}}{k_{i}(k_{i}-1)}$ Where $t_{i}$ is the number of triangles around node i , and $k_{i}$ is its degree.
Shortest path length	Quantifies the average shortest distance from a given node to all other nodes in the network, indicating routing efficiency.	$L_{i}=\frac{1}{N-1}\sum_{j\neq i\in G}d_{\mathrm{ij}}$
Nodal efficiency	Characterizes the efficiency of a given node in parallel information transfer across the network.	$E_{\text{nodal},i}=\frac{1}{N-1}\sum_{j\neq i\in G}\frac{1}{d_{\mathrm{ij}}}$
Degree centrality	Reflects the number of connections a node has, representing the strength of its capacity for information exchange.	$k_{i}=\sum_{j\in G}a_{\mathrm{ij}}$
Betweenness centrality	Indicates how often a node lies on the shortest paths between other node pairs, revealing its control role in information flow.	$b_{i}=\sum_{h\neq j,h\neq i,j\neq i\in G}\frac{\rho h_{j}(i)}{\rho h_{j}}$ Where $\rho_{hj}$ is the total number of shortest paths between h and j , and $\rho_{hj}(i)$ is the number of those paths that pass through node i .

To comprehensively evaluate the group differences and ensure that the findings were not driven by the selection of a single arbitrary threshold, statistical analyses were performed repeatedly at each individual sparsity threshold (ranging from 0.05 to 0.50). First, an Analysis of Covariance (ANCOVA) with age as a covariate was employed to assess the main effects of the group on the network topological metrics. Statistical significance was defined at a *p* < 0.05. Finally, the resulting *p*-values across all thresholds were extracted and visualized as continuous heatmaps, which allowed us to robustly demonstrate the distribution and stability of significant topological differences across the entire density spectrum.

Node-level topological metrics primarily include clustering coefficient, shortest path length, nodal efficiency, degree centrality, and betweenness centrality. Among them, the clustering coefficient is used to measure the degree of interconnection among nodes within the neighborhood of a given node; the shortest path length reveals its routing efficiency in the network; node efficiency reflects its role in local network information integration and transmission; degree centrality serves as a fundamental indicator of a node’s ability to exchange information; betweenness centrality highlights its pivotal role in global information flow.

In order to avoid the deviation caused by a single threshold, a certain range of sparsity threshold is adopted in this study. Sparsity is defined as the ratio of the number of edges actually reserved in a network to the maximum possible number of edges in a fully connected network. The sparsity threshold range of this study is set to 0.05 to 0.50, and the step size is 0.01.

## 3 Research results

### 3.1 Analysis of structural brain connectivity using the NBS method

**Figure 2:**
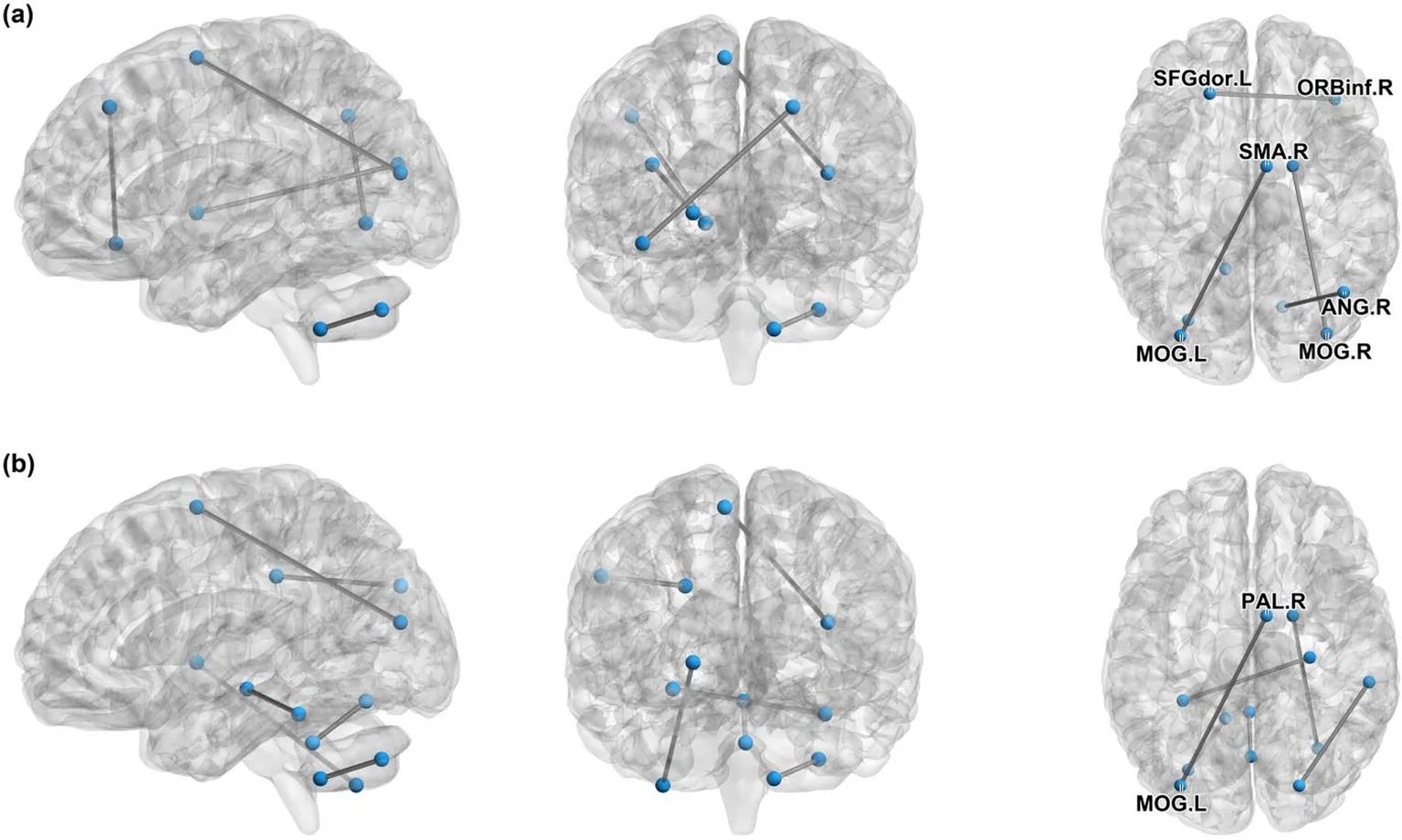
Structural connectivity differences between the elite and second-level groups **(a)** Connections stronger in the elite group than in the second-level group; **(b)** Connections stronger in the second-level group than in the elite group. Selective node labels are shown on the axial views to improve readability, while sagittal and coronal views are kept unlabeled.

**Figure 3:**
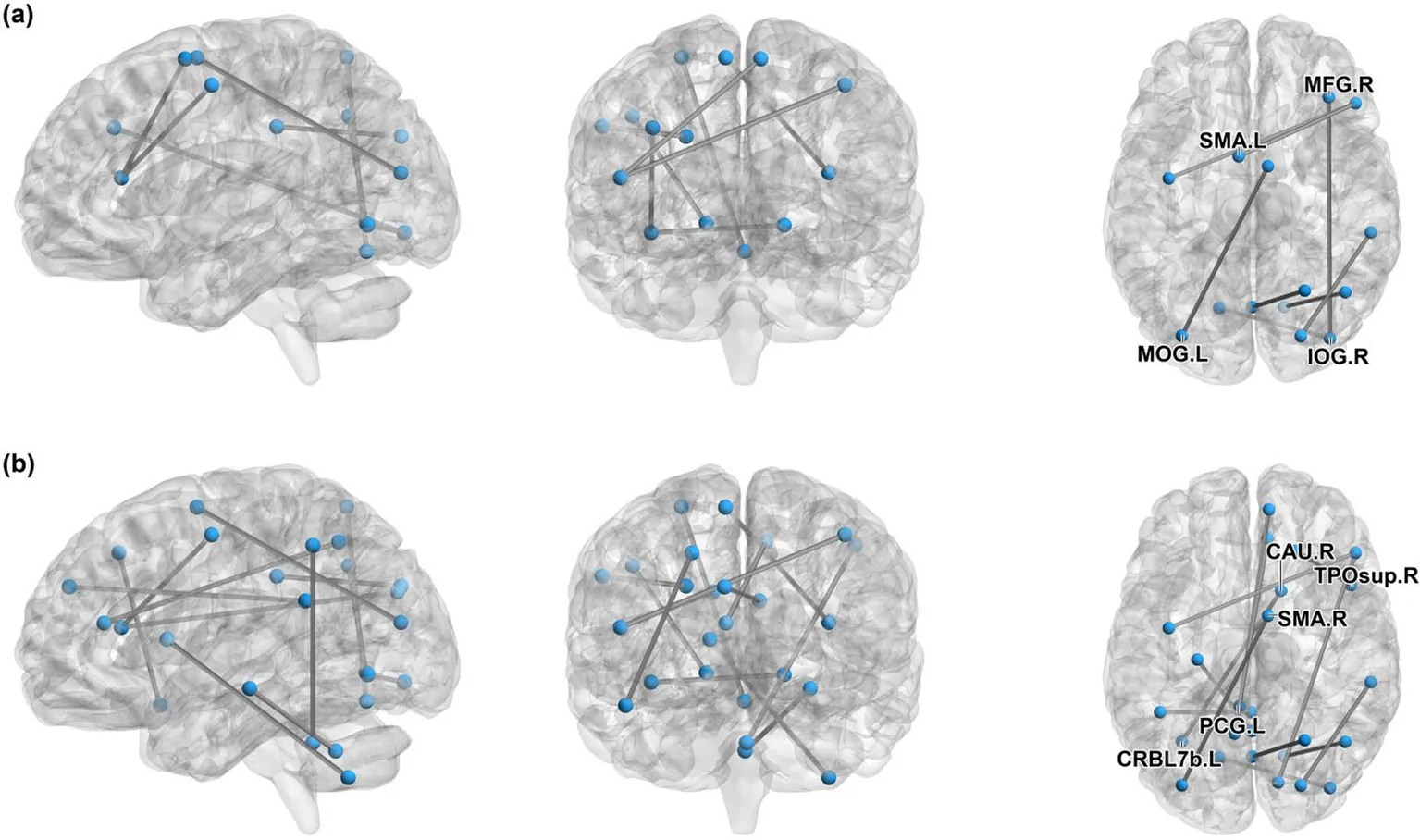
Structural connectivity differences between the elite and ordinary groups. **(a)** Connections stronger in the elite group than in the ordinary group; **(b)** Connections stronger in the ordinary group than in the elite group. Selective node labels are shown on the axial views to improve readability, while sagittal and coronal views are kept unlabeled.

**Figure 4:**
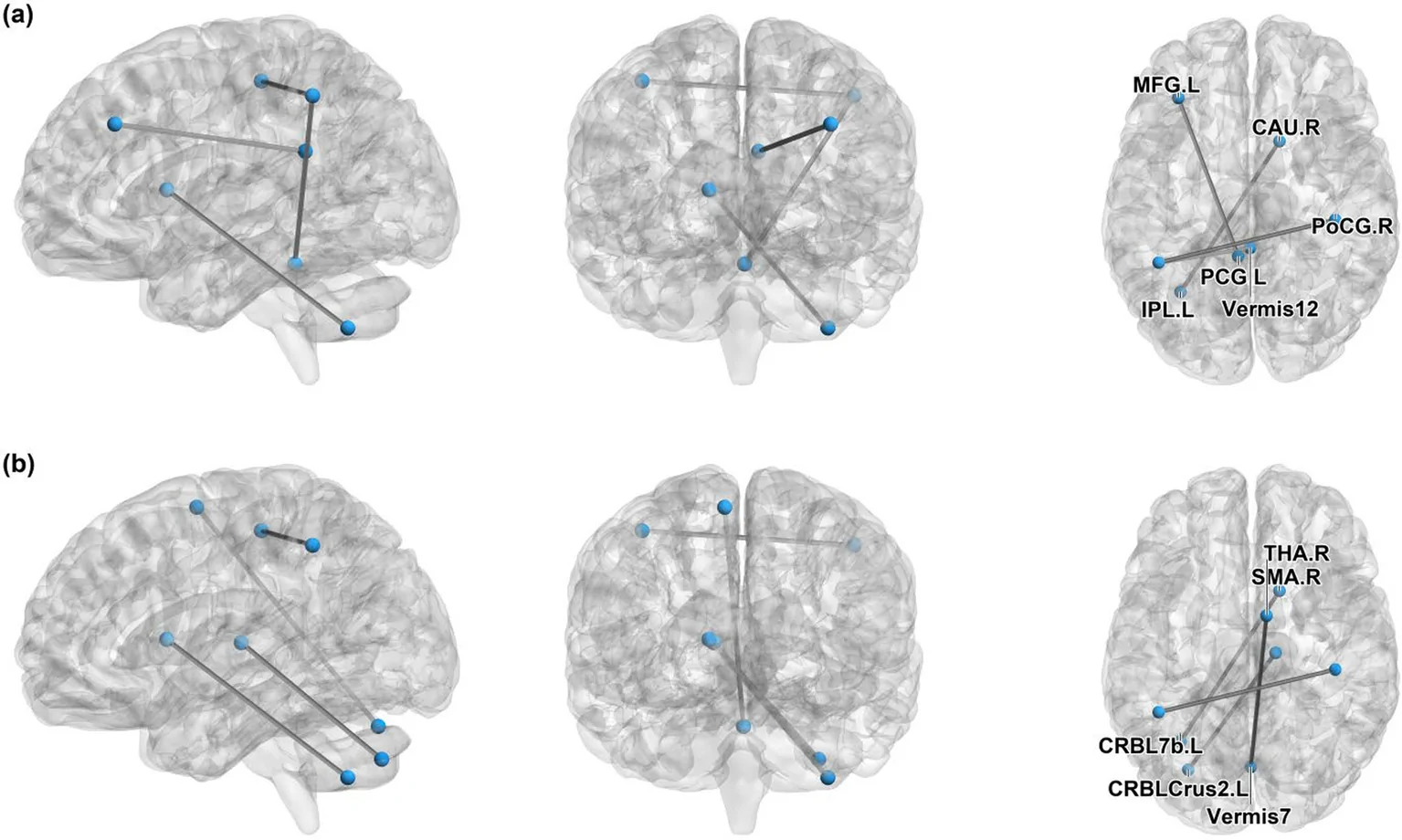
Structural connectivity differences between the second-level and ordinary groups. **(a)** Connections stronger in the second-level group than in the ordinary group; **(b)** Connections stronger in the ordinary group than in the second-level group. Selective node labels are shown on the axial views to improve readability, while sagittal and coronal views are kept unlabeled.

**Figure 5:**
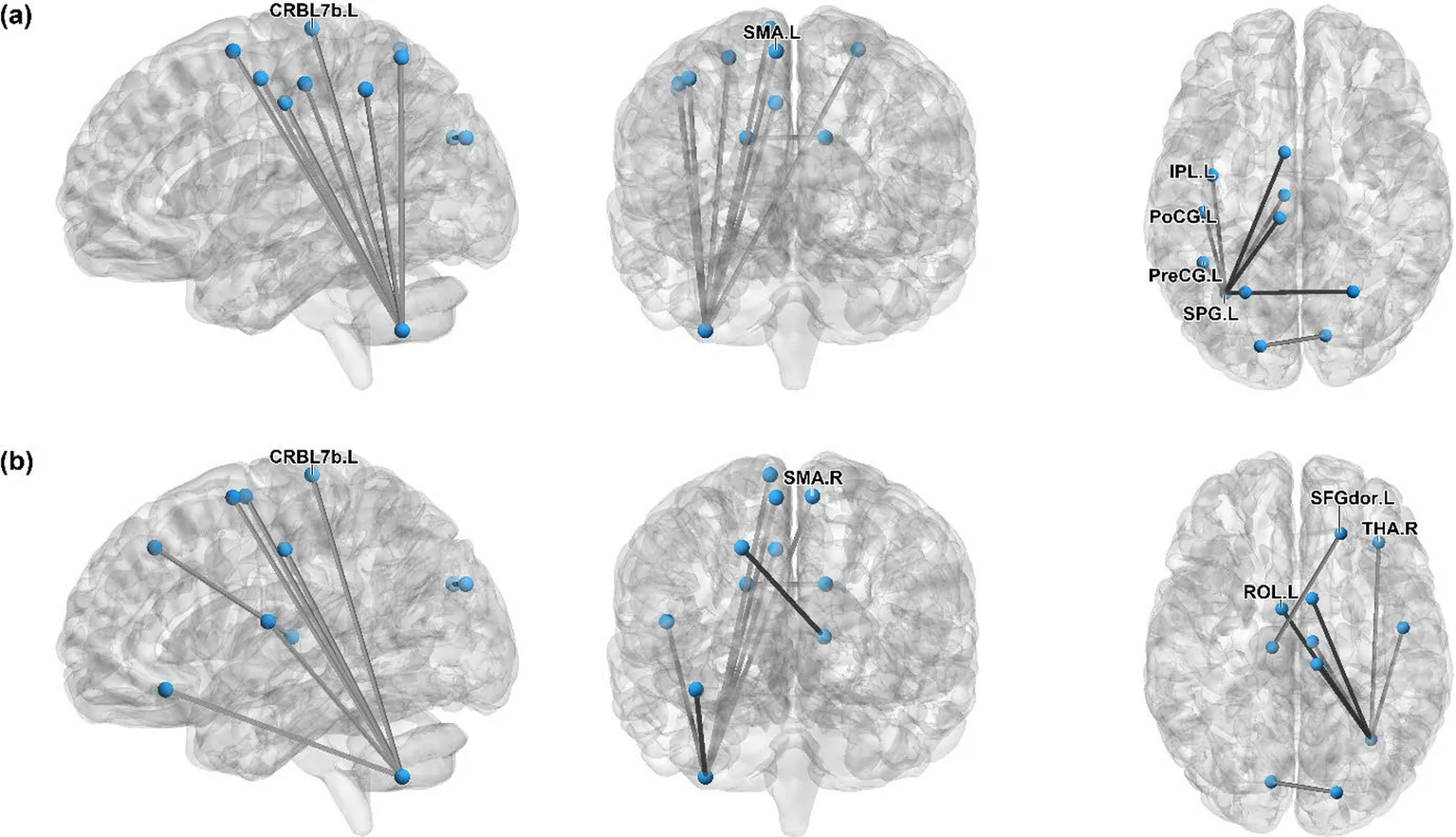
Functional connectivity differences between the elite and second-level groups. **(a)** Connections stronger in the elite group than in the second-level group; **(b)** connections stronger in the second-level group than in the elite group. Selective node labels are shown on the axial views to improve readability, while sagittal and coronal views are kept unlabeled.

**Figure 6:**
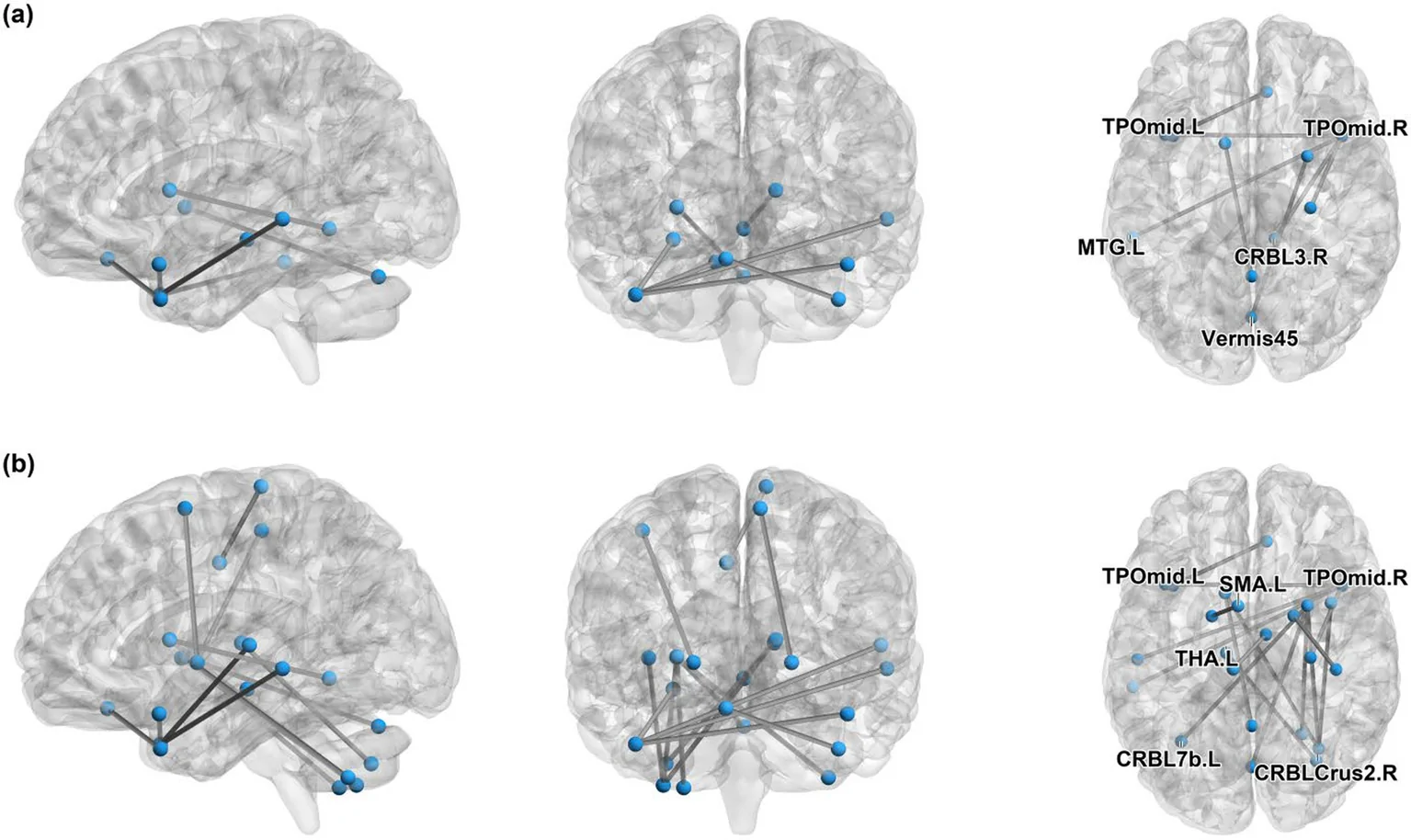
Functional connectivity differences between the elite and ordinary groups. **(a)** Connections stronger in the elite group than in the ordinary group; **(b)** Connections stronger in the ordinary group than in the elite group. Selective node labels are shown on the axial views to improve readability, while sagittal and coronal views are kept unlabeled.

**Figure 7:**
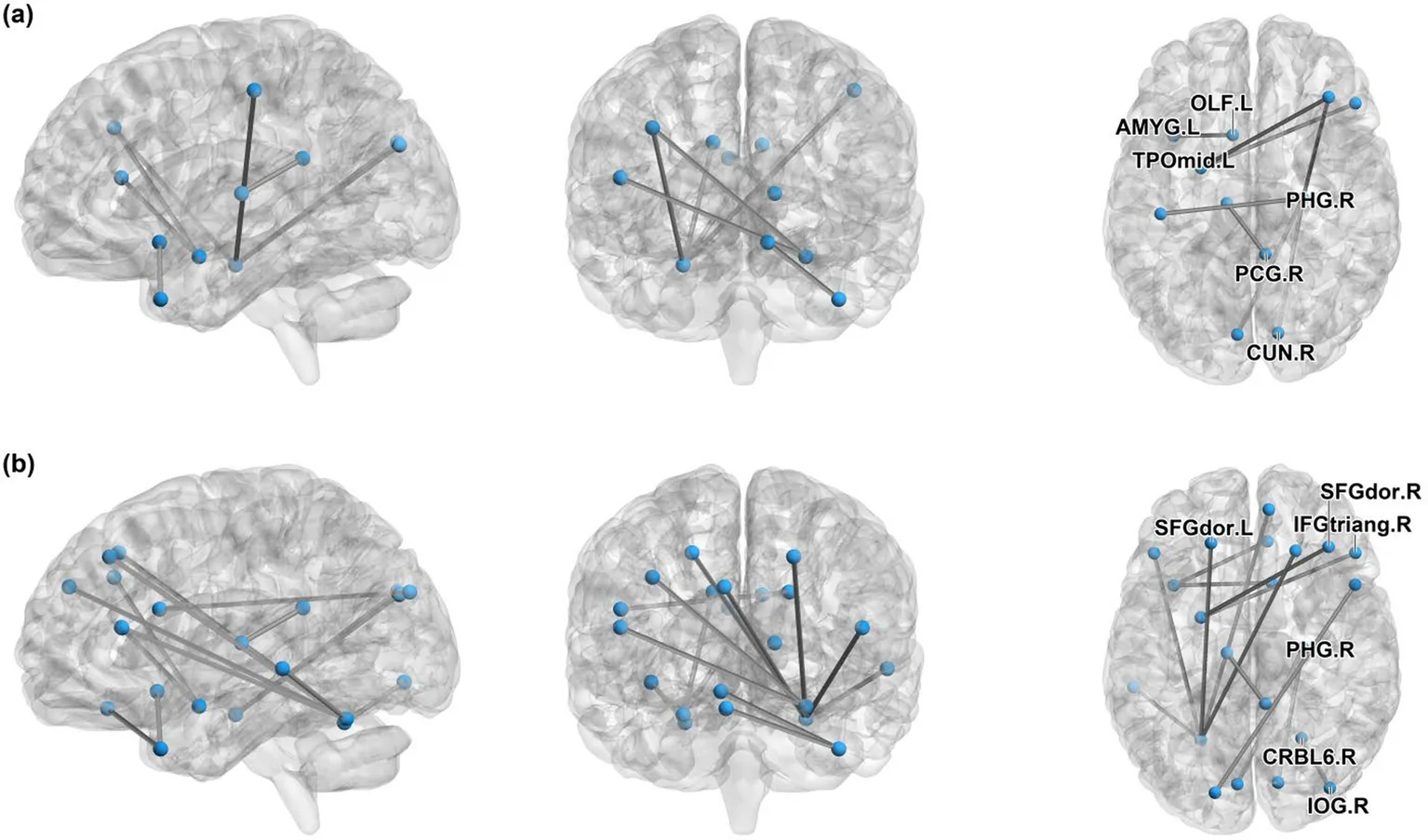
Functional connectivity differences between the second-level and ordinary groups. **(a)** Connections stronger in the second-level group than in the ordinary group; **(b)** Connections stronger in the ordinary group than in the second-level group. Selective node labels are shown on the axial views to improve readability, while sagittal and coronal views are kept unlabeled.

**Figure 8:**
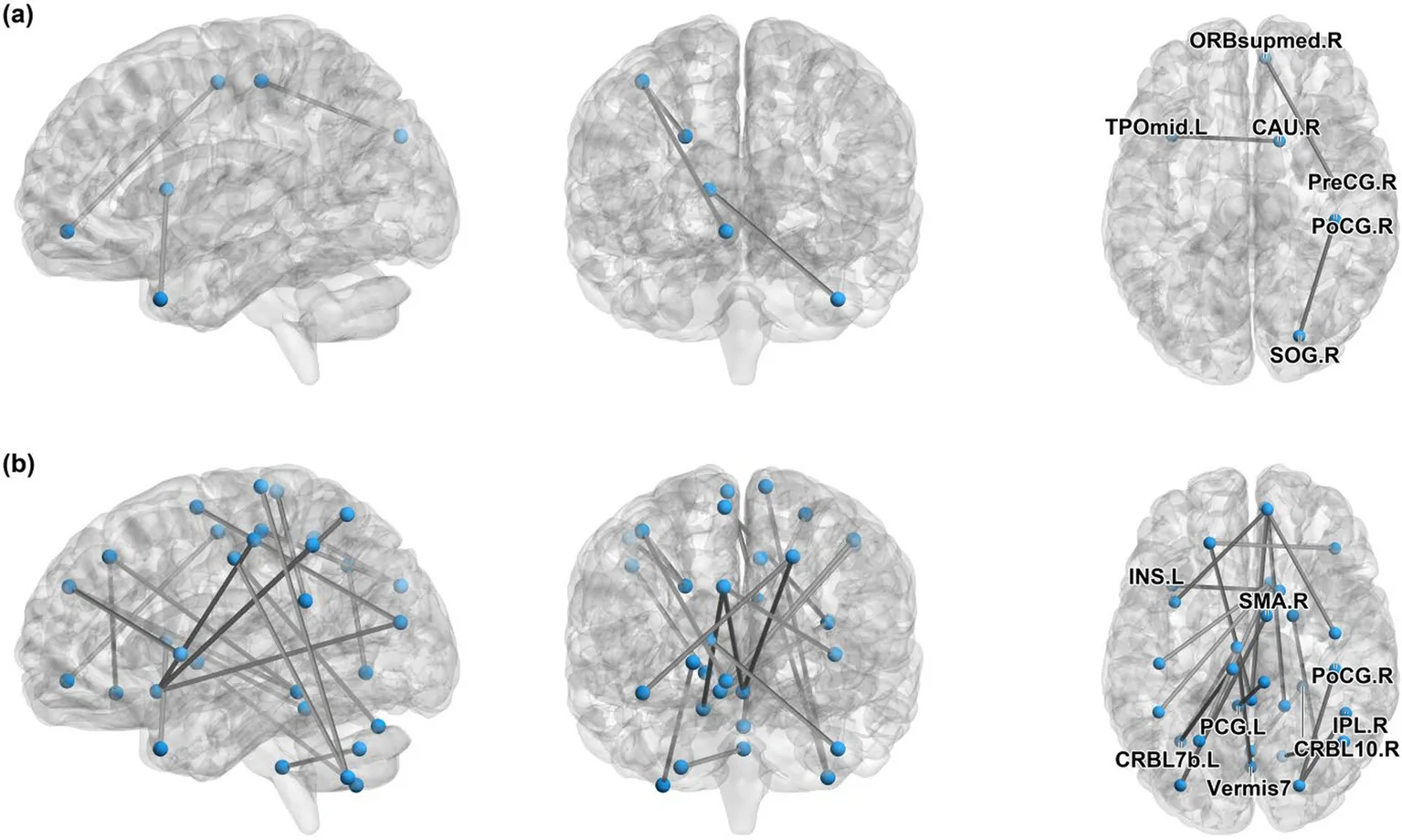
Structure–function coupling differences between the elite and second-level groups. **(a)** connections stronger in the elite group than in the second-level group; **(b)** connections stronger in the second-level group than in the elite group. Selective node labels are shown on the axial views to improve readability, while sagittal and coronal views are kept unlabeled.

**Figure 9:**
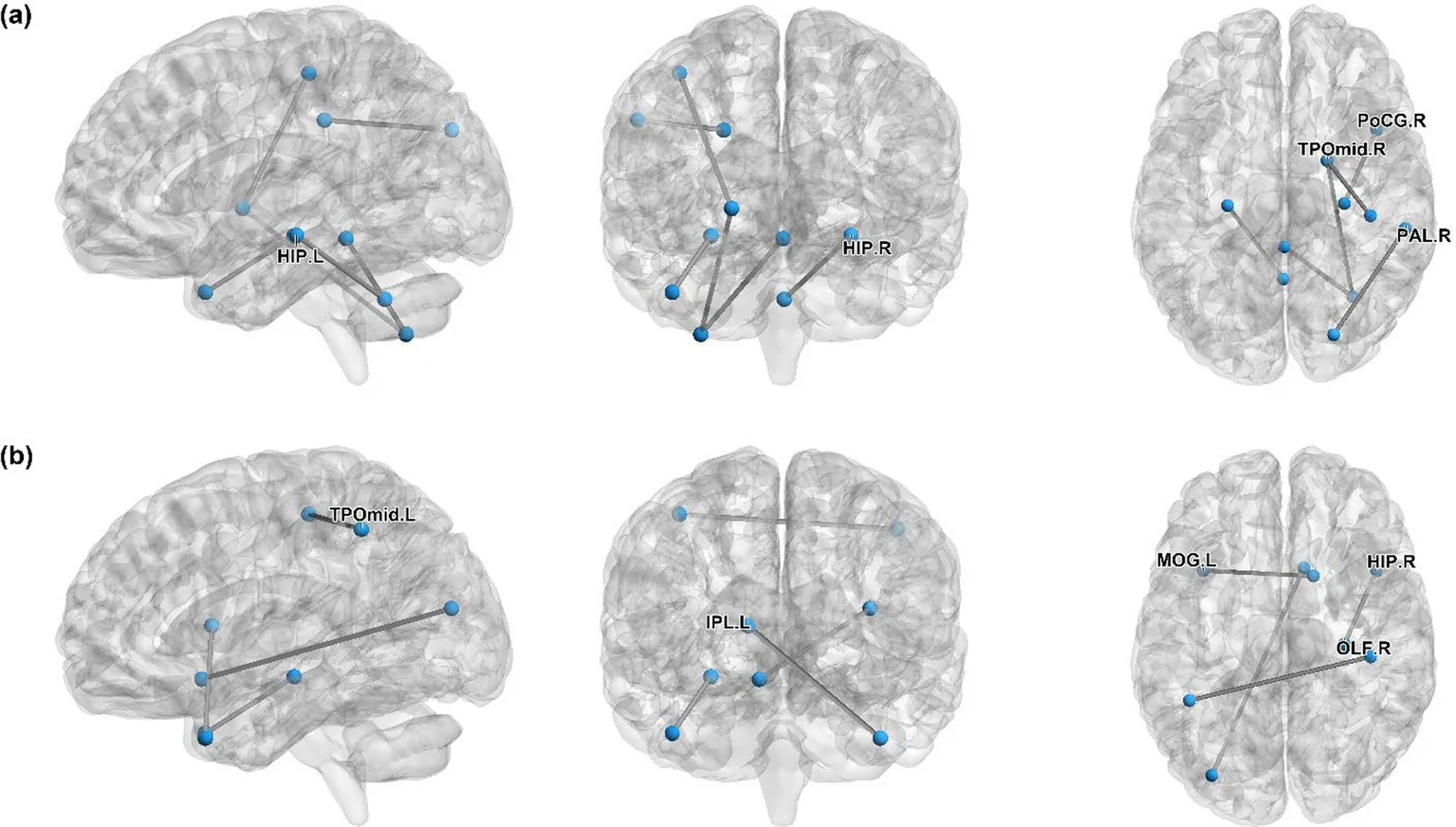
Structure–function coupling differences between the elite and ordinary groups. **(a)** Connections stronger in the elite group than in the ordinary group; **(b)** Connections stronger in the ordinary group than in the elite group. Selective node labels are shown on the axial views to improve readability, while sagittal and coronal views are kept unlabeled.

**Figure 10:**
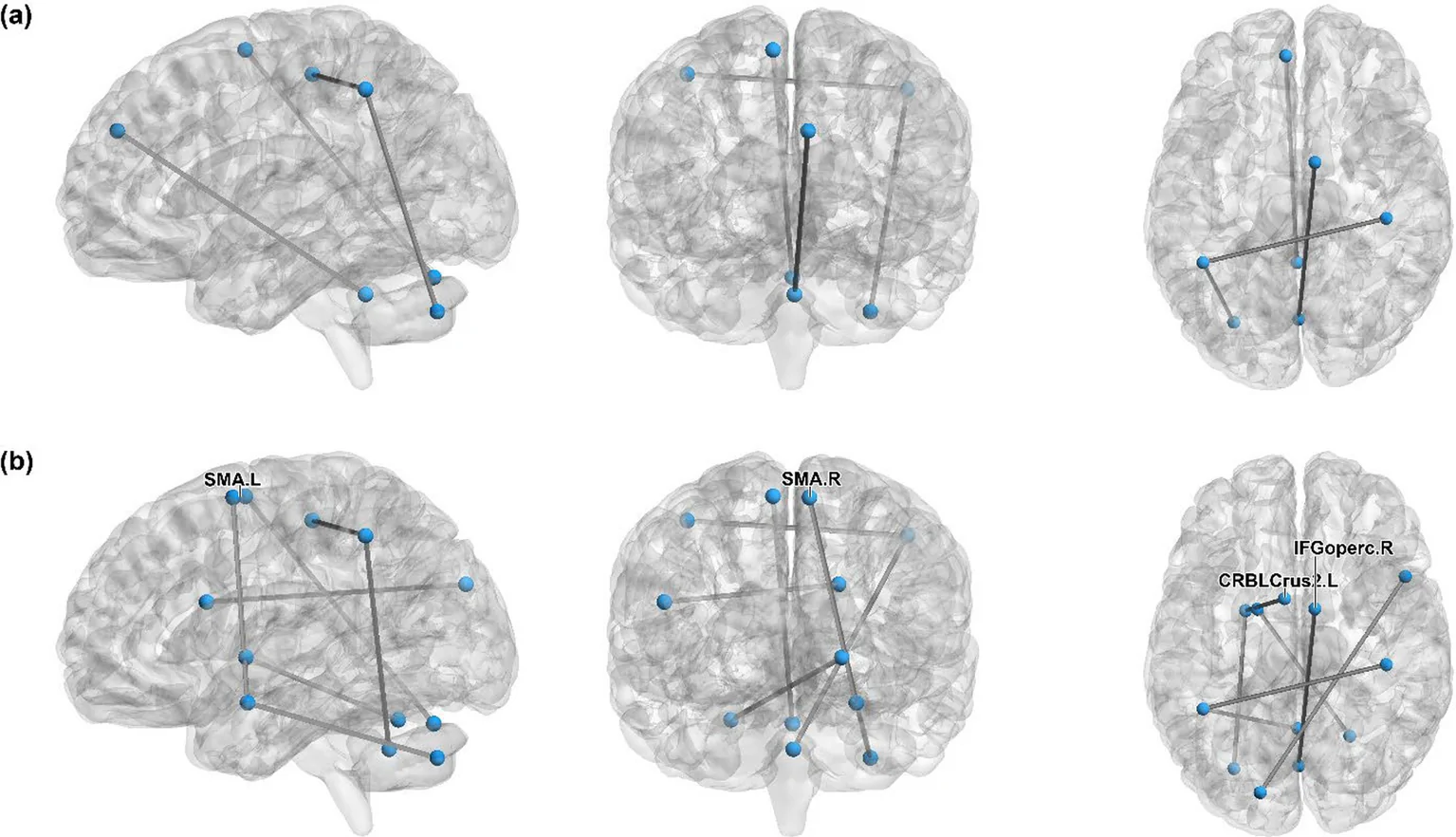
Structure–function coupling differences between the second-level and ordinary groups. **(a)** Connections stronger in the second-level group than in the ordinary group; **(b)** Connections stronger in the ordinary group than in the second-level group. Selective node labels are shown on the axial views to improve readability, while sagittal and coronal views are kept unlabeled.

In this section, the NBS method was used to conduct a comparative analysis of the brain structural connectivity characteristics among different subject groups. By comparing the structural connection matrices across different groups, identify subnetworks that exhibit statistically significant differences. Multiple comparisons were corrected at the network level using a permutation-based FWER procedure, with a significance threshold of *p* < 0.05. In Figures 2–10, selected principal nodes are directly annotated with anatomical abbreviations on the axial views to improve visual interpretability while minimizing label overlap.

#### 3.1.1 Comparison between elite group and second-level group

As shown in Figure 2, compared to secondary-level athletes, the elite group exhibited stronger structural connectivity in regions such as the SFGdor.L (dorsolateral superior frontal gyrus), SMA.R (supplementary motor area), ORBinf.R (orbital part of the inferior frontal gyrus), MOG.L/R (middle occipital gyrus), and ANG.R (angular gyrus). In contrast, the second-level group showed stronger connectivity in regions including the LING.R (lingual gyrus) and PAL.R (lenticular nucleus, pallidum).

#### 3.1.2 Comparison between elite group and ordinary group

As shown in Figure 3, the principal labeled regions in the connections stronger in the elite group included MFG.R (right middle frontal gyrus), SMA.L (left supplementary motor area), MOG.L (left middle occipital gyrus), and IOG.R (right inferior occipital gyrus). The principal labeled regions in the connections stronger in the ordinary group included CAU.R (right caudate nucleus), TPOsup.R (right temporal pole of the superior temporal gyrus), SMA.R (right supplementary motor area), PCG.L (left posterior cingulate gyrus), and CRBL7b.L (left cerebellar lobule VIIb).

#### 3.1.3 Comparison between second-level group and ordinary group

As shown in Figure 4, compared to the ordinary group, the other group exhibited enhanced structural connectivity primarily involving the MFG.L(Middle frontal gyrus), PCG.L (Posterior cingulate gyrus), IPL.L(Inferior parietal lobule), PCC.L, CAU.R, PoCG.R(Postcentral gyrus), and Vermis12. These regions primarily belong to the frontal–parietal control network and the cortical–basal ganglia–cerebellar circuit. The ordinary group showed stronger connections in regions such as cerebellar Crus2.L, CRBL7b.L, Vermis7, SMA.R, and THA.R, forming a cerebellar-cortical motor integration pathway.

In summary, the NBS analysis results indicate that Sanda athletes with different levels of athletic performance exhibit significant differences in brain structural connectivity patterns. The differences in the elite athlete group are mainly concentrated in the frontal–parietal-occipital pathways and cerebellum-related regions.

### 3.2 Analysis of brain functional connectivity characteristics based on the NBS method

This section uses the NBS method to compare and analyze the brain functional connectivity characteristics of different subject groups. By comparing the functional connectivity matrices between groups, statistically significant subnetworks were identified. To correct for multiple comparisons, a permutation-based FWER procedure was applied at the network level, with the significance threshold set at *p* < 0.05.

#### 3.2.1 Comparison between elite group and second-level group

As shown in Figure 5, the enhanced connections in the elite group are mainly concentrated in areas such as CRBL7b.L, SMA.L, IPL.L, PoCG.L, PreCG.L (Precentral gyrus), SPG.L (Superior parietal gyrus), PCL.L, SOG.L (Superior occipital gyrus), and DCG.L (Median cingulate and paracingulate gyri), belonging to SMN and DAN. In contrast, the enhanced connections in the second-level group are concentrated in CRBL7b.L, SMA.R, THA.R, SFGdor.L, ROL.L (Rolandic operculum), and ORBinf.L, forming the cerebellum-thalamus-frontal lobe pathway and executive control network (ECN).

#### 3.2.2 Comparison between elite group and ordinary group

As shown in Figure 6, the principal labeled regions in the connections stronger in the elite group included TPOmid.L/R (bilateral temporal poles of the middle temporal gyri), MTG.L (left middle temporal gyrus), CRBL3.R (right cerebellar lobule III), and Vermis45 (cerebellar vermis IV-V). The principal labeled regions in the connections stronger in the ordinary group included SMA.L (left supplementary motor area), THA.L (left thalamus), TPOmid.L/R, CRBL7b.L (left cerebellar lobule VIIb), and CRBLCrus2.R (right cerebellar Crus II).

#### 3.2.3 Comparison between the second-level group and ordinary group

As shown in Figure 7, the principal labeled regions in the connections stronger in the second-level group included AMYG.L (left amygdala), OLF.L (left olfactory cortex), TPOmid.L (left temporal pole of the middle temporal gyrus), PHG.R (right parahippocampal gyrus), PCG.R (right posterior cingulate gyrus), and CUN.R (right cuneus). The principal labeled regions in the connections stronger in the ordinary group included SFGdor.L/R (bilateral dorsolateral superior frontal gyri), IFGtriang.R (right triangular part of the inferior frontal gyrus), PHG.R, CRBL6.R (right cerebellar lobule VI), and IOG.R (right inferior occipital gyrus).

Overall, the NBS functional connectivity results revealed differential network patterns across groups with different levels of athletic performance. The elite athlete group relied more on the collaboration of SMN, DAN, the limbic system, and DMN.

### 3.3 Analysis of brain structure–function coupling connectivity characteristics based on the NBS method

This section employs the NBS method to conduct a comparative analysis of brain structure–function coupling characteristics across different subject groups. By comparing the structure–function coupling matrices between groups, sub-networks with statistically significant differences were identified. During the analysis, FWER correction was applied at the network level, with a significance threshold set at *p* < 0.05. In this study, the SC-FC linear combination is used as an operational multimodal coordination index rather than a direct biophysical model of either white-matter microstructure or BOLD synchronization alone. The resulting graph metrics therefore quantify the relative topological alignment between anatomical connectivity and functional co-fluctuation patterns under a predefined weighting scheme. Accordingly, these SFC metrics should be interpreted as indicators of group-level multimodal network organization, not as direct measures of a single neurobiological substrate. This value was used as a predefined operational parameter to introduce functional connectivity information while retaining the dominant contribution of the structural connectivity backbone. Given the absence of a universally accepted weighting parameter for linear SC–FC integration, the choice of α should be interpreted as a methodological setting rather than a biologically optimized parameter.

#### 3.3.1 Comparison between elite group and second-level group

As shown in Figure 8A, the enhanced coupling in the elite athletes group mainly involves the TPOmid.L (temporal–parietal-occipital junction), ORBsupmed.R (superior medial orbitofrontal cortex), CAU.R (caudate nucleus), PreCG/PoCG.R (precentral/postcentral gyrus), and SOG.R (superior occipital gyrus). These regions are part of ECN, SMN, and visual system. Figure 8B shows that the enhanced coupling in the secondary athletes group is primarily distributed across CRBL7b.L, CRBL10.R, Vermis (cerebellum), SMA.R, PoCG.R, INS.L (insula), IPL.R (inferior parietal lobule), and PCC.L (posterior cingulate cortex). These regions are involved in the cerebellar-cortical circuits, the ventral attention network (VAN), and DMN.

#### 3.3.2 Comparison between elite group and ordinary group

As shown in Figure 9A, the enhanced coupling in the elite athletes group is primarily concentrated in HIP.L/R (bilateral hippocampus), PoCG.R, SMG.R (supramarginal gyrus), TPOmid.R, and PAL.R (globus pallidus), involving the limbic system, SMN, and ECN. Figure 9B shows that the enhanced coupling in the general university student group is distributed across TPOmid.L, IPL.L, MOG.L, HIP.R, OLF.R, and CAU.R, which belong to DMN, the visual system, and the cortical-basal ganglia motor circuits.

#### 3.3.3 Comparison between the second-level group and ordinary group

As shown in Figure 10A, the enhanced coupling in the secondary athletes group primarily includes TPOmid.L, THA.L (thalamus), OLF.L (olfactory bulb), AMYG.L (amygdala), PCG.R/PHG.R (cingulate gyrus/parahippocampal gyrus), MFG.R (middle frontal gyrus), and IFGtriang.R (inferior frontal gyrus, triangular part). These regions form the limbic system–prefrontal control circuit. Figure 10B shows that the enhanced coupling in the general university student group involves SMA.L/R, CRUS2.L/R (cerebellar cortex), and IFGoperc.R (inferior frontal gyrus opercular part), primarily belonging to the SMN, cerebellar network, and motor control circuits.

In summary, the SFC analysis results reveal the differences in structure–function coupling patterns across individuals with different levels of athletic ability. The elite athletes group shows stronger cross-network integration (ECN, SMN, limbic system); the secondary athletes group is in a transitional state; the general university student group relies more on the DMN, visual, and basic motor circuits.

### 3.4 Analysis of brain structural network topological properties based on GRETNA

This section utilizes the GRETNA tool to compute graph-theoretical metrics for the SC matrices of the three groups, encompassing both global and node-level network topological indicators. By analyzing both global and local information, the differences in brain structural network organization across individuals with varying levels of athletic ability are examined. Covariance analysis is subsequently performed on each metric, controlling for covariates such as age, with FDR correction applied for multiple comparisons. Significant differences in the metrics are further visualized to elucidate the potential impact of training background on brain structural network characteristics.

**Figure 11:**
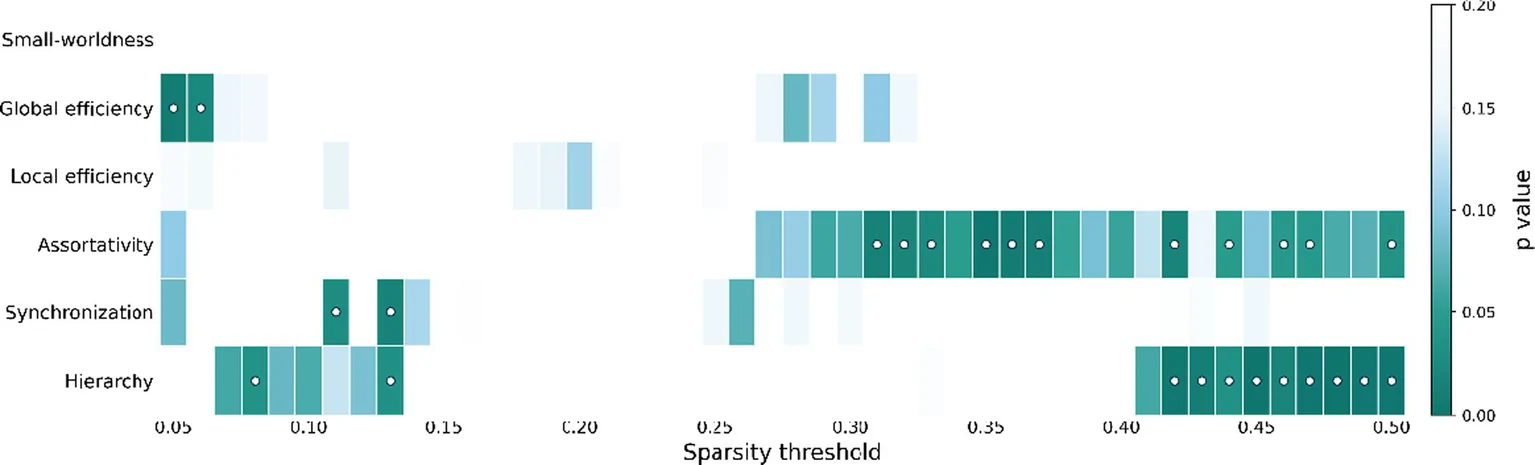
Heatmap of *p*-values derived from ANCOVA for global topological metrics of the structural network across a range of sparsity thresholds. Columns represent sparsity thresholds (0.05–0.50), and rows correspond to small-worldness, global efficiency, local efficiency, assortativity, synchronization, and hierarchy. The color gradient indicates the *p*-values. White dots inside the cells indicate statistically significant main effects among the groups (*p <* 0.05). Age was included as a covariate in the statistical model.

Figure 11 illustrates the significant changes in global metrics at different sparsity thresholds. The results indicate that small-world properties and local efficiency did not show significant differences across the three groups. However, global efficiency, assortativity, synchrony, and hierarchy exhibited trends of difference at certain thresholds.

**Figure 12:**
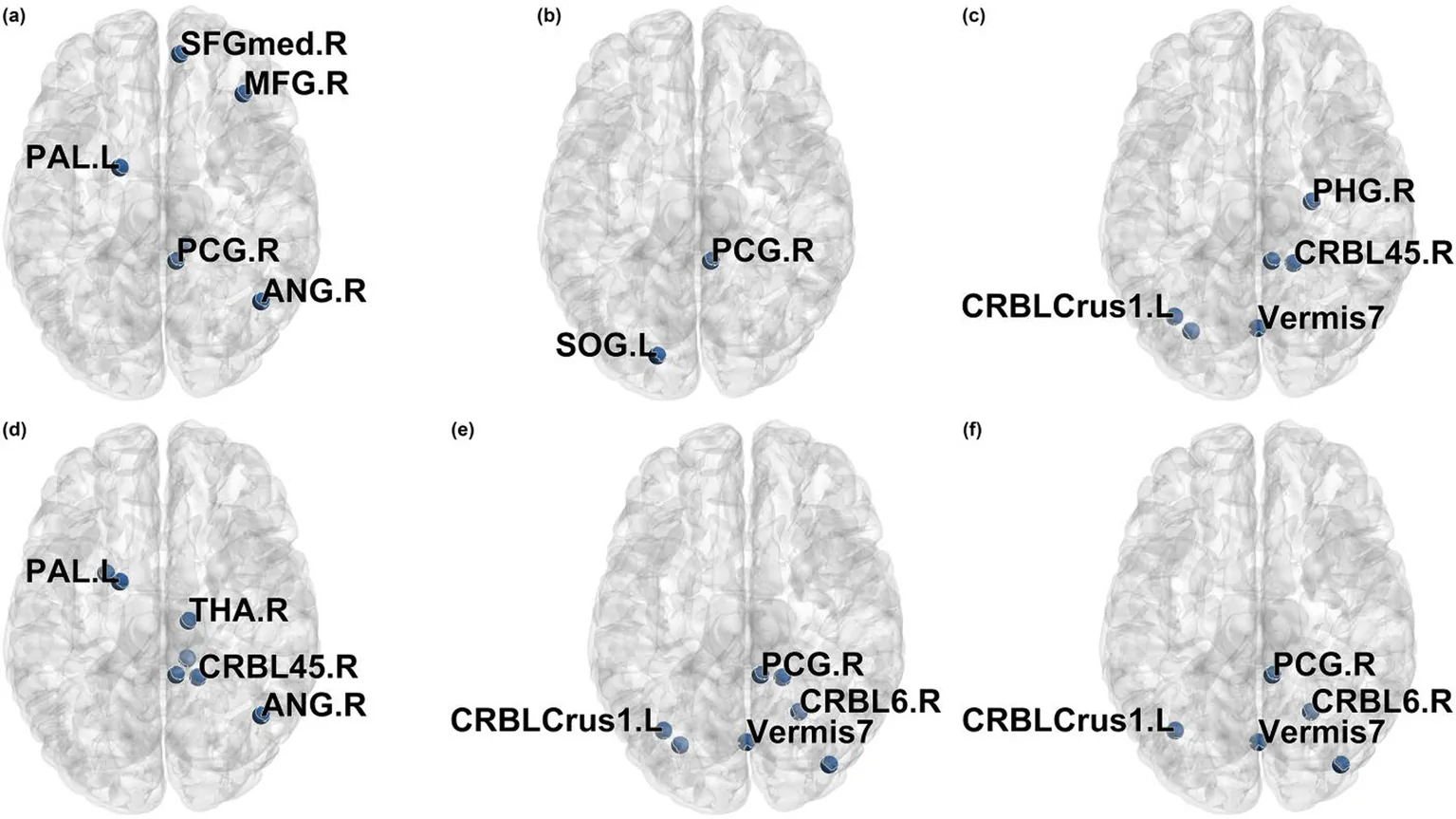
Visualized results of the node-level structural network metrics for the elite, secondary, and ordinary groups under different sparsity thresholds. **(a)** Shows the clustering coefficient, **(b)** shows the shortest path length, **(c)** shows nodal efficiency, **(d)** shows nodal local efficiency, **(e)** shows degree centrality, and **(f)** shows betweenness centrality. The nodes shown in the figure represent the core brain regions with statistically significant differences between groups. In order to minimize visual interference, all significant nodes are visualized with uniform size and color, aiming at clearly indicating their spatial anatomical positions. Bold node labels were retained, and closely spaced labels were selectively displayed to improve readability while preserving the visible node locations.

Figure 12 illustrates the distribution of node-level differences. The results show that these differences are mainly concentrated in key hub regions, including the frontoparietal areas, supramarginal gyrus, cingulate gyrus, and cerebellar cortex. Specifically, the elite athletes group exhibits significantly increased degree centrality and nodal efficiency in nodes such as SMG.R and CRBL6.R. The secondary athletes group shows enhanced metrics in several frontal and parietal nodes (e.g., IPL and MFG).

In summary, the graph-theoretical analysis based on GRETNA further reveals that individuals with different levels of athletic ability exhibit differences in the global integration efficiency and local key nodes of brain structural networks.

### 3.5 Analysis of brain functional network topological properties based on GRETNA

In this study, the GRETNA tool was used to compute graph-theoretical metrics at both global and node levels for the functional connectivity matrices of the three groups. At different sparsity thresholds, covariance analysis was first conducted to control for covariates such as age.

**Figure 13:**
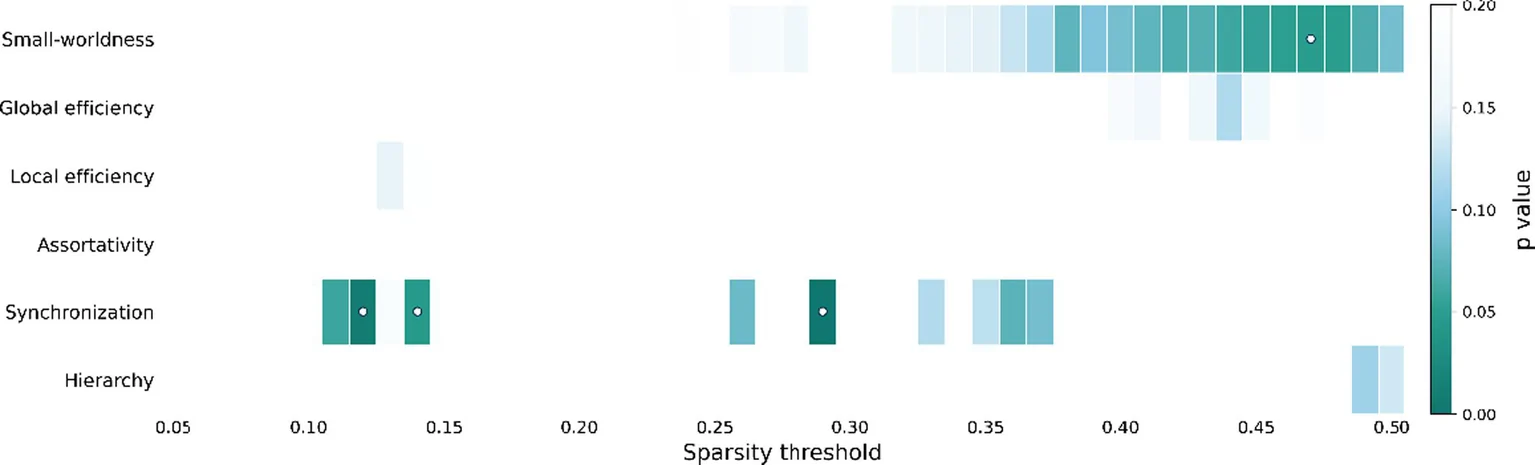
Heatmap of *p*-values derived from ANCOVA for global topological metrics of the functional network across a range of sparsity thresholds. Columns represent sparsity thresholds (0.05–0.50), and rows correspond to small-worldness, global efficiency, local efficiency, assortativity, synchronization, and hierarchy. The color gradient indicates the *p*-values. White dots inside the cells indicate statistically significant main effects among the groups (*p* < 0.05). Age was included as a covariate in the statistical model.

The results show that, as illustrated in Figure 13, at the global level, small-worldness exhibited significant group differences at a threshold of 0.47, while synchrony showed significant differences at thresholds of 0.12, 0.14, and 0.29. No significant differences were observed for the other metrics, including global efficiency, local efficiency, assortativity, and hierarchy, across the range of thresholds.

**Figure 14:**
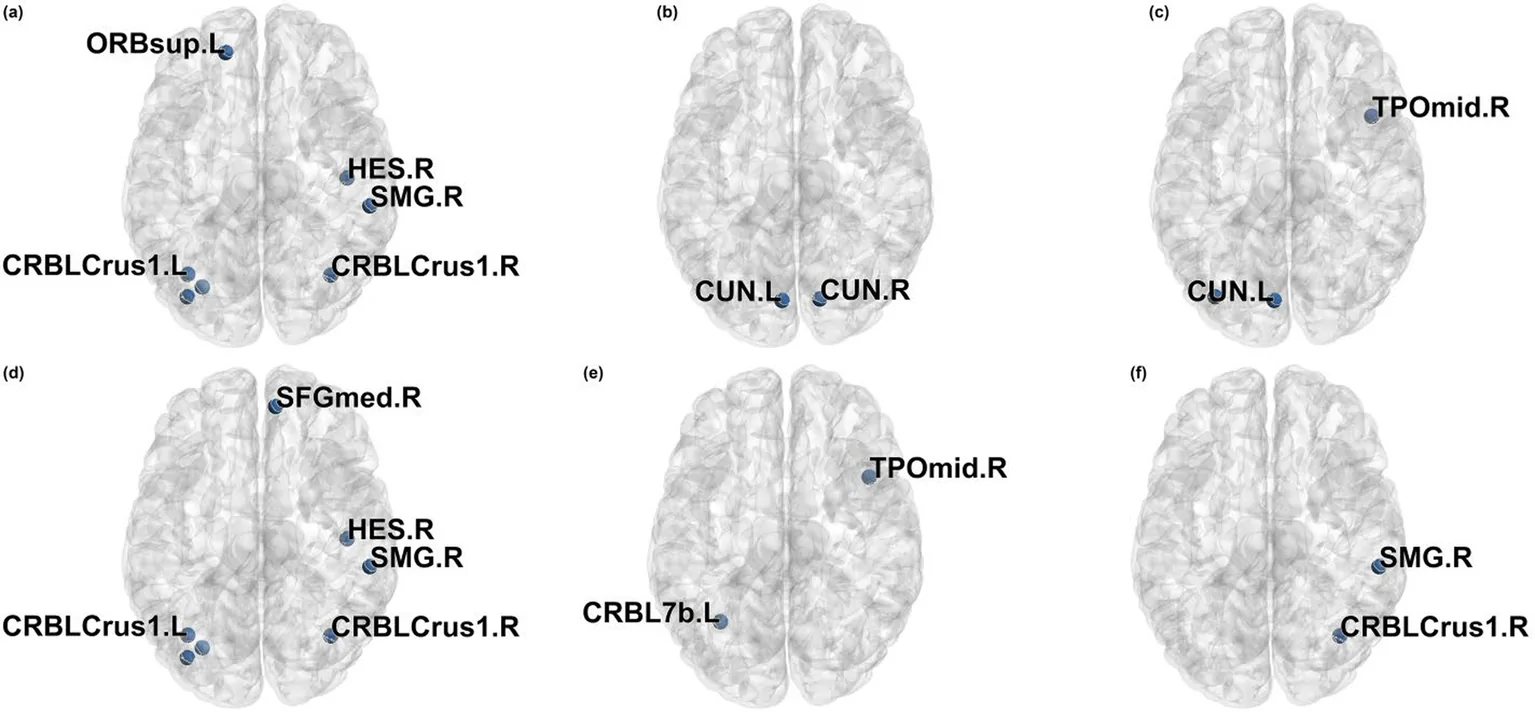
Visualized results of the node-level functional network metrics for the elite, secondary, and ordinary groups under different sparsity thresholds. **(A)** shows clustering coefficient, **(B)** shows shortest path length, **(C)** shows nodal efficiency, **(D)** shows nodal local efficiency, **(E)** shows degree centrality, and **(F)** shows betweenness centrality. Bold node labels were retained, and closely spaced labels were selectively displayed to improve readability while preserving the visible node locations. The nodes shown in the figure represent the core brain regions with statistically significant differences between groups. In order to minimize visual interference, all significant nodes are visualized with uniform size and color, aiming at clearly indicating their spatial anatomical positions.

At the node level, as shown in Figure 14, significant differences were primarily distributed across the parietal-cerebellar network, visual cortex, and temporal association areas. Specifically, the clustering coefficient was significantly elevated in regions such as the ORBsup.L (Orbital part of superior frontal gyrus), HES.R, SMG.R, and CRUS1/2. For nodal local efficiency, degree centrality, and betweenness centrality, the cerebellar CRUS regions and SMG.R were repeatedly identified.

In summary, the results of the functional network topological analysis indicate that Sanda athletes with different levels of athletic ability exhibit differences at both global and local levels. The elite athletes group demonstrates advantages in small-worldness and synchrony. At the nodal level, the reorganization of key hub regions, including the cerebellum, parietal, and frontal areas, further highlights the deep shaping of sensorimotor and cognitive control networks induced by long-term systematic training.

### 3.6 Analysis of brain structure–function coupling network topological properties based on GRETNA

In this study, the GRETNA tool was further employed to compute graph-theoretical metrics at both global and node levels for SFC matrices of the three groups. At different sparsity thresholds, covariance analysis was conducted to control for covariates such as age, followed by FDR correction to ensure the robustness and scientific rigor of the results.

**Figure 15:**
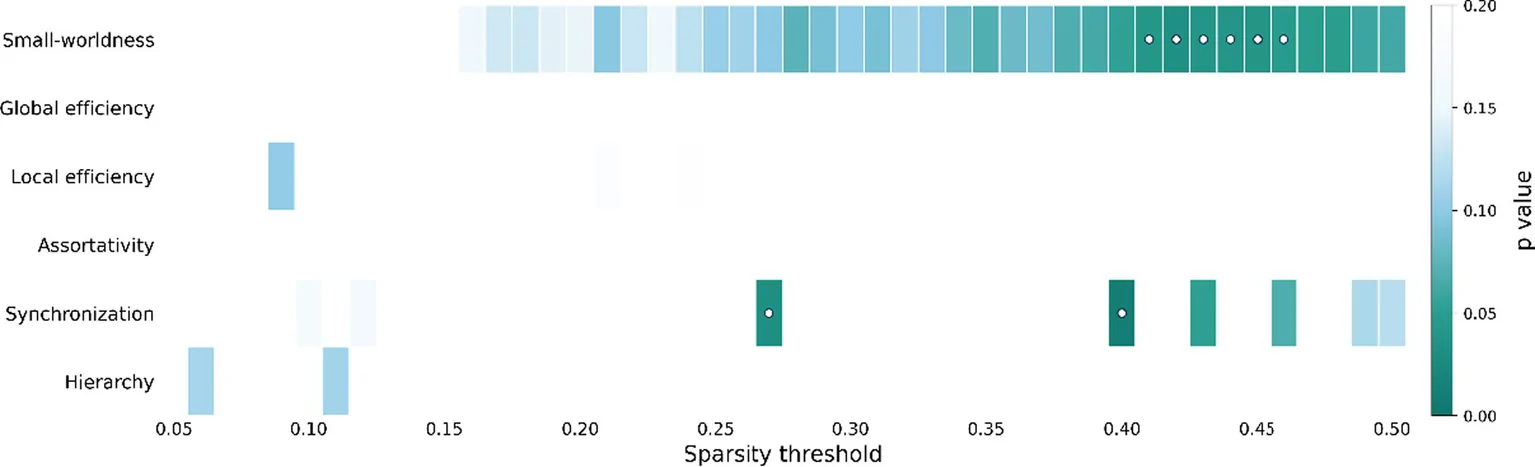
Heatmap of *p*-values derived from ANCOVA for global topological metrics of the structure–function coupling network across a range of sparsity thresholds. Columns represent sparsity thresholds (0.05–0.50), and rows correspond to small-worldness, global efficiency, local efficiency, assortativity, synchronization, and hierarchy. The color gradient indicates the *p*-val*ues. White dots inside the cells indicate statistically significant main effects among the group*s (*p* < 0.05). Age was included as a covariate in the statistical model.

As shown in Figure 15, at the global level, small-worldness exhibited stable and significant differences across the threshold range of 0.41 to 0.46, consistent with previous findings that athletic training can enhance the balance between segregation and integration in brain networks(Fuchs and Fluegge, 2014; Yan et al., 2024). In addition, synchrony also showed significant differences at thresholds of 0.27 and 0.40. No statistically significant differences were observed for the other metrics, including global efficiency, local efficiency, assortativity, and hierarchy, across all thresholds.

**Figure 16:**
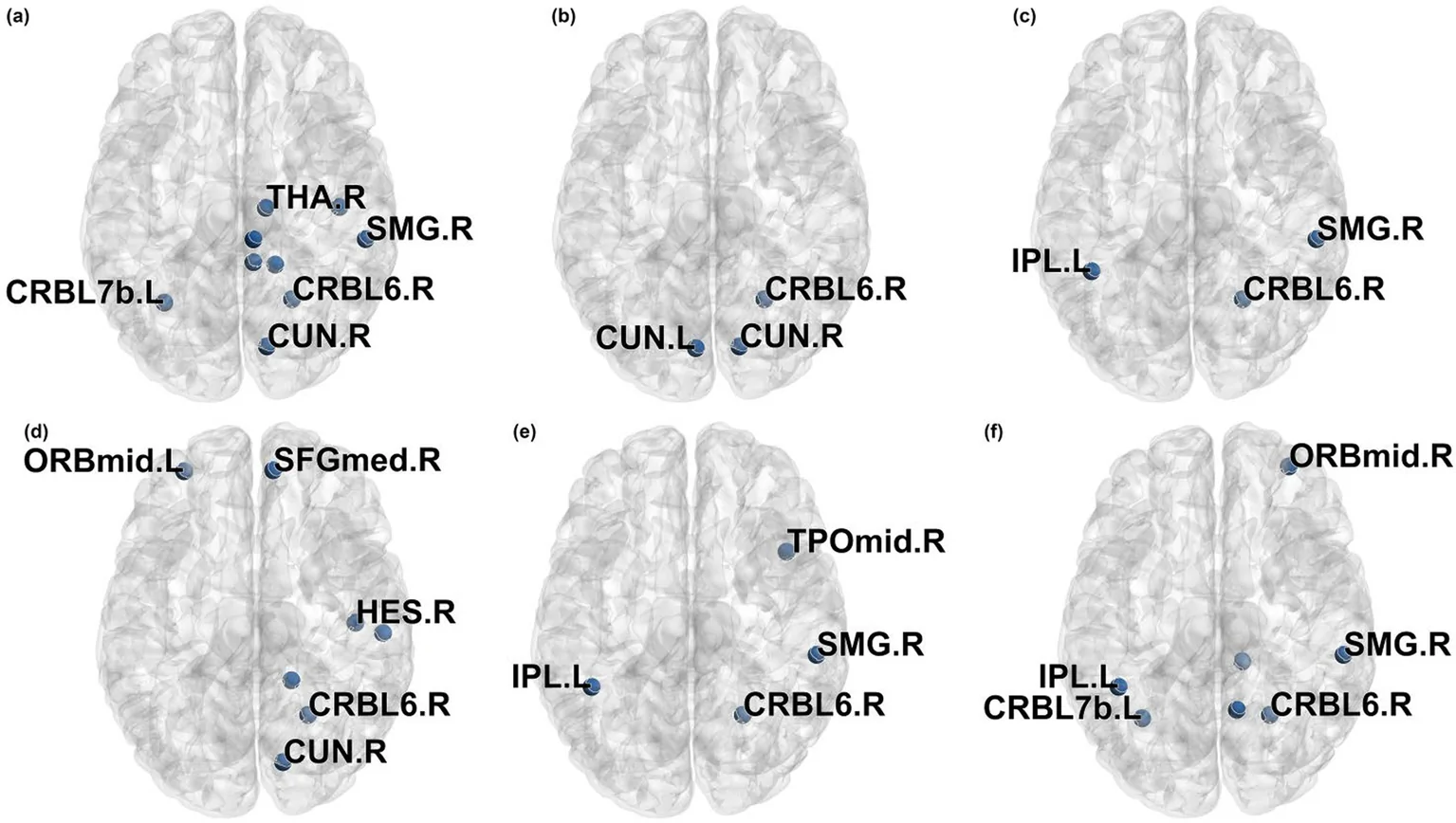
Visualized results of the node-level structure–function coupling metrics for the elite, secondary, and ordinary groups under different sparsity thresholds. **(A)** shows clustering coefficient, **(B)** shows shortest path length, **(C)** shows nodal efficiency, **(D)** shows nodal local efficiency, **(E)** shows degree centrality, and **(F)** shows betweenness centrality. Note: The nodes shown in the figure represent the core brain regions with statistically significant differences between groups. In order to minimize visual interference, all significant nodes are visualized with uniform size and color, aiming at clearly indicating their spatial anatomical positions. Bold node labels were retained, and closely spaced labels were selectively displayed to improve readability while preserving the visible node locations.

At the node level, as shown in Figure 16, significant differences were primarily concentrated within the parietal–frontal–cerebellar network. For metrics such as clustering coefficient, nodal local efficiency, and degree centrality, SMG.R, IPL.L, and CRBL6.R were repeatedly identified. In addition, regions showing significant differences in shortest path length, nodal efficiency, and betweenness centrality included CUN, PCUN, and THA.R, which are located at the intersection of the default mode network and parieto-posterior pathways.

## 4 Discussion

To better interpret the present findings, the multimodal results are more informatively understood from a network-driven rather than a method-driven perspective. Instead of considering the NBS, global graph-theoretical, and nodal graph-theoretical findings separately, the convergent pattern across SC, FC, and SFC suggests that long-term Sanda training reshapes several interconnected neurobiological systems (Petersen and Sporns, 2015). In particular, four functionally meaningful networks appear to account for the major training-related differences observed in the present study: the parietal-cerebellar sensorimotor loop, the fronto-parietal executive control network, the limbic-basal ganglia-cerebellar circuit, and the visual-sensorimotor integration network. Together, these systems support rapid perception, decision making, motor planning, and adaptive execution in highly dynamic combat environments (Petersen and Sporns, 2015; Zhang et al., 2021; Yan et al., 2024).

### 4.1 Parietal-cerebellar sensorimotor loop

The parietal-cerebellar loop emerged as the most consistent adaptive network across modalities. At the structural level, NBS analysis showed that training-related differences repeatedly involved cerebellar regions together with parietal and sensorimotor areas, suggesting strengthened anatomical pathways for movement coordination and online motor adjustment (Park et al., 2015; Zhang et al., 2021). At the functional level, cerebellar and parietal regions also appeared within the differential subnetworks, indicating more efficient integration of somatosensory, vestibular, and motor information (Yan et al., 2024; Wang J. et al., 2025). At the coupling level, the repeated involvement of SMG, IPL, PoCG, and cerebellar subdivisions such as CRBL6 and CRBL7b suggests tighter coordination between structural constraints and functional dynamics within this loop (Zhang et al., 2021).

In addition, graph-theoretical analysis further emphasized the importance of this system. At the nodal level, SMG.R and CRBL6.R were repeatedly identified as key regions with enhanced centrality and efficiency, indicating that they function as important hubs for network integration. At the global level, the training-related advantages in small-worldness and synchrony are also consistent with a more stable and efficient sensorimotor coordination architecture (Petersen and Sporns, 2015; Wang J. et al., 2025). Functionally, this loop is highly relevant to rapid perception-action coupling, postural adjustment, rhythm regulation, and precise motor timing, all of which are essential for offensive-defensive transitions in Sanda (Manni and Petrosini, 2004; Grimm et al., 2025). Collectively, these findings suggest that long-term Sanda training reinforces the parietal-cerebellar sensorimotor loop, thereby supporting fast and accurate action selection in complex competitive contexts (Park et al., 2015; Zhang et al., 2021; Wang J. et al., 2025).

### 4.2 Fronto-parietal executive control network

A second major pattern involved the fronto-parietal executive control network. Structurally, elite and second-level athletes showed altered connectivity in frontal and parietal regions such as MFG, SFG, IPL, PCC, and related association areas, indicating experience-dependent strengthening of pathways relevant to executive regulation (Zhang et al., 2021). Functionally, frontal regions including SFGdor and orbitofrontal areas were also implicated in the NBS results, suggesting that training affects the dynamic coordination of attention allocation, inhibitory control, and action planning (Marek et al., 2015; Wendelken et al., 2016). At the SFC level, the co-involvement of frontal, parietal, and cingulate regions further supports the idea that high-level performance depends on the alignment of anatomical organization with flexible control-related functional interactions (Petersen and Sporns, 2015; Zhang et al., 2021).

This interpretation is also supported by the topological findings. Several frontal and parietal nodes showed enhanced nodal properties, suggesting greater hub-like roles in information transfer and regulatory efficiency. Although not all global metrics were consistently significant, the observed differences in small-worldness and synchrony still point to a more optimized large-scale organizational framework that may facilitate rapid decision making under pressure (Marek et al., 2015; Petersen and Sporns, 2015). In neurobiological terms, the fronto-parietal control system likely supports attentional shifting, tactical evaluation, response inhibition, and context-dependent action selection (Wendelken et al., 2016; Wang J. et al., 2025). Therefore, the present results suggest that long-term Sanda training does not simply improve motor execution, but also promotes more efficient executive control over perception and behavior (Marek et al., 2015; Zhang et al., 2021).

### 4.3 Limbic-basal ganglia-cerebellar circuit

The limbic-basal ganglia-cerebellar circuit represents a third important adaptive system identified in the present study. At the SC level, elite athletes showed differential connectivity involving subcortical and limbic-related regions such as CAU, PUT, HIP, and cerebellar structures, suggesting stronger anatomical support for motor program selection, procedural memory, and context-sensitive behavioral regulation (Gong et al., 2019). At the FC level, regions including AMYG, PHG, thalamus, and cerebellar areas were implicated in the differential subnetworks, indicating that training may also reshape the functional coordination between affective, mnemonic, and motor systems (Zhang et al., 2021; Yan et al., 2024). At the SFC level, the repeated appearance of hippocampal, caudate, orbitofrontal, and cerebellar regions further suggests that successful performance depends on stable coupling between emotion-related, memory-related, and action-related systems (Zhang et al., 2021).

This circuit is especially meaningful in the context of Sanda, which requires not only motor skill, but also emotional regulation, action readiness, and rapid retrieval of learned tactical patterns. The basal ganglia contribute to action selection and sequence optimization, the hippocampal-limbic system supports contextual memory and strategic experience, and the cerebellum refines timing and coordination (Manni and Petrosini, 2004; Gong et al., 2019). When considered together, these findings suggest that higher-level Sanda performance is associated with stronger integration across emotion, memory, and motor-control systems, allowing athletes to maintain stable execution while adapting to rapidly changing competitive demands (Fuchs and Fluegge, 2014; Yan et al., 2024).

### 4.4 Visual-sensorimotor integration network

The visual-sensorimotor integration network constitutes a fourth key component of training-related neuroplasticity. Across the NBS results, occipital and visual association regions such as MOG, LING, CUN, SOG, and IOG repeatedly appeared together with parietal and motor-related areas. Structurally, this pattern suggests enhanced pathways linking visual input systems with motor planning and execution regions (Park et al., 2015). Functionally, altered connectivity within occipital, parietal, and cerebellar regions indicates more efficient transformation of external sensory information into coordinated motor responses (Tan et al., 2017; Yan et al., 2024). At the SFC level, the simultaneous involvement of visual, parietal, and sensorimotor nodes further indicates that visual information processing is closely coupled with action control in trained athletes (Zhang et al., 2021).

The corresponding nodal findings support this interpretation. Regions such as CUN, IOG, SMG, and related sensorimotor areas showed prominent nodal differences, suggesting enhanced local processing efficiency and relay capacity. For Sanda athletes, such adaptations are particularly important because successful performance depends on accurately detecting opponent movement, rapidly estimating spatial relationships, and transforming visual cues into appropriate offensive or defensive actions (Petersen and Sporns, 2015; Tan et al., 2017). Therefore, the present results indicate that long-term Sanda training promotes a tighter integration between perceptual systems and motor-control systems, providing a neural basis for fast visuomotor transformation and adaptive behavior in complex competitive environments (Park et al., 2015; Zhang et al., 2021; Yan et al., 2024).

### 4.5 Integrative summary

Taken together, the present findings suggest that the neuroplastic effects of long-term Sanda training are best characterized as the coordinated reorganization of multiple interacting brain systems rather than isolated changes detected by separate analytical methods. The parietal-cerebellar loop appears to provide the core substrate for rapid sensorimotor integration and movement calibration; the fronto-parietal network supports executive regulation and tactical control; the limbic-basal ganglia-cerebellar circuit contributes to emotionally stable and experience-guided action selection; and the visual-sensorimotor network enables efficient conversion of perceptual information into action. The convergent evidence across SC, FC, SFC, and graph-theoretical topology therefore supports a coherent neurobiological account in which high-level Sanda performance depends on the progressive integration of sensorimotor, executive, affective, and perceptual systems (Park et al., 2015; Petersen and Sporns, 2015; Zhang et al., 2021; Yan et al., 2024).

A methodological consideration is that the present sample included only male participants, which may limit the generalizability of these network-level adaptation patterns to female Sanda athletes. Future studies should therefore examine whether the same hierarchical organization of training-related neuroplasticity is preserved across sexes.

## 5 Conclusion

This study employed the NBS method and the graph-theoretical analysis tool GRETNA to systematically investigate the network characteristics of brain structural connectivity, functional connectivity, and structure–function coupling in individuals with different training levels (elite athletes, secondary athletes, and general university students). Based on the construction of connectivity matrices using the AAL116 atlas and between-group comparisons of neuroimaging data from 35 participants, the following conclusions were drawn:

1. Differential Subnetworks: NBS analysis identified multiple subnetworks showing significant differences at the structural, functional, and structure–function coupling levels. Elite athletes exhibited pronounced coupling in visual–motor–executive pathways, whereas the ordinary group showed characteristic enhancements in language- and rhythm-control-related pathways.
2. Global and Node-Level Advantages: Graph-theoretical metrics indicated that elite athletes outperformed the other groups in small-worldness and certain key nodal properties, such as centrality and efficiency, suggesting more optimized information transmission and integration within their brain networks. This advantage aligns closely with the neural demands of Sanda-specific techniques, including rapid transitions between offense and defense, continuous combinations, and the coordination of striking and throwing in high-intensity competitive scenarios.
3. Functional Module Adaptation: Differential connections were primarily concentrated in the sensorimotor network, fronto–executive network, and cerebellar regulatory systems, indicating targeted adaptive reorganization of brain networks across different training levels. In Sanda-specific training, elite athletes require rapid visual–motor matching, instantaneous switching of motor programs, and cerebellar–cortical regulation for postural control, all of which are reflected in the network differences revealed in this study.

Overall, this study is the first to reveal, within the context of competitive Wushu, the shaping effects of long-term Sanda training on brain connectivity and topological properties from a multi-level network perspective. The findings indicate that the high-level neural regulation exhibited by Sanda athletes in the integrated perception–decision–execution chain and striking–throwing composite techniques is closely associated with adaptive reorganization of brain networks in small-world organization, synchrony coordination, and cross-modal integration. Future studies could further combine brain network characteristics with biomechanical metrics of Sanda-specific movements—such as offensive and defensive rhythms, striking combinations, and throwing techniques—to systematically elucidate the neural mechanisms induced by Sanda training, thereby providing theoretical and practical guidance for training optimization, skill enhancement, and rehabilitation interventions.

## Data Availability

The original contributions presented in the study are included in the article/Supplementary material, further inquiries can be directed to the corresponding author/s.
