# Supplementary material for: Brain network topology and neural mechanisms in Sanda athletes: a comparative study across training levels
Source: Front Neurosci. 2026 Sep 4;20:1801936. doi: 10.3389/fnins.2026.1801936 (PMC13587693; doi:10.3389/fnins.2026.1801936)
Supplement: Supplementary file 1 [file Data_Sheet_1.DOCX]

**Supplementary Material**

This single document contains all supplementary items cited in the manuscript: Supplementary Figure S1 and Supplementary Tables S1-S2.

**Supplementary Figure S1**

**Alpha sensitivity analysis for structure-function coupling construction.**

The figure summarizes the robustness of SFC-related group-difference patterns across alpha values ranging from 0.1 to 0.9. Pairwise t-statistic similarity, edge-set overlap, top-ranked edge overlap, and the number of supra-threshold edges are shown relative to the predefined alpha = 0.3 setting.

**Supplementary Table S1**

**Summary of alpha sensitivity analysis for SFC construction.**

This table summarizes the range of pairwise t-statistic correlations, edge-set overlap, top-10 edge overlap, and number of significant edges across alpha values from 0.1 to 0.9, relative to the predefined alpha = 0.3 setting.

| **Comparison** | **Correlation range** | **Jaccard range** | **Top-10 overlap** | **Significant edges** | **Edges at alpha = 0.3** |
| --- | --- | --- | --- | --- | --- |
| G1 vs G2 | 0.656-1.000 | 0.143-1.000 | 4-10 | 1-6 | 2 |
| G1 vs G3 | 0.652-1.000 | 0.143-1.000 | 3-10 | 2-6 | 2 |
| G2 vs G3 | 0.675-1.000 | 0.000-1.000 | 2-10 | 1-6 | 1 |

Abbreviations: G1, elite group; G2, second-level group; G3, ordinary group. Correlations and overlaps are calculated relative to alpha = 0.3.

**Supplementary Table S2**

**Detailed alpha sensitivity results across alpha values.**

This table reports the number of significant edges, correlation with alpha = 0.3, Jaccard overlap of significant edge sets, and top-10 edge overlap for each group comparison at each tested alpha value.

| **Alpha** | **Comparison** | **Significant edges** | **Correlation vs 0.3** | **Jaccard vs 0.3** | **Top-10 overlap** |
| --- | --- | --- | --- | --- | --- |
| 0.1 | G1 vs G2 | 1 | 0.954 | 0.500 | 9 |
| 0.1 | G1 vs G3 | 3 | 0.949 | 0.667 | 9 |
| 0.1 | G2 vs G3 | 1 | 0.936 | 1.000 | 10 |
| 0.2 | G1 vs G2 | 2 | 0.990 | 1.000 | 10 |
| 0.2 | G1 vs G3 | 2 | 0.989 | 1.000 | 10 |
| 0.2 | G2 vs G3 | 1 | 0.986 | 1.000 | 10 |
| 0.3 | G1 vs G2 | 2 | 1.000 | 1.000 | 10 |
| 0.3 | G1 vs G3 | 2 | 1.000 | 1.000 | 10 |
| 0.3 | G2 vs G3 | 1 | 1.000 | 1.000 | 10 |
| 0.4 | G1 vs G2 | 2 | 0.990 | 1.000 | 10 |
| 0.4 | G1 vs G3 | 3 | 0.990 | 0.667 | 8 |
| 0.4 | G2 vs G3 | 3 | 0.988 | 0.333 | 6 |
| 0.5 | G1 vs G2 | 3 | 0.958 | 0.667 | 9 |
| 0.5 | G1 vs G3 | 3 | 0.958 | 0.667 | 8 |
| 0.5 | G2 vs G3 | 3 | 0.952 | 0.333 | 6 |
| 0.6 | G1 vs G2 | 3 | 0.904 | 0.250 | 7 |
| 0.6 | G1 vs G3 | 2 | 0.903 | 0.333 | 8 |
| 0.6 | G2 vs G3 | 5 | 0.897 | 0.200 | 4 |
| 0.7 | G1 vs G2 | 6 | 0.831 | 0.143 | 4 |
| 0.7 | G1 vs G3 | 3 | 0.828 | 0.250 | 6 |
| 0.7 | G2 vs G3 | 5 | 0.827 | 0.200 | 3 |
| 0.8 | G1 vs G2 | 6 | 0.744 | 0.143 | 4 |
| 0.8 | G1 vs G3 | 3 | 0.741 | 0.250 | 6 |
| 0.8 | G2 vs G3 | 6 | 0.750 | 0.167 | 2 |
| 0.9 | G1 vs G2 | 6 | 0.656 | 0.143 | 4 |
| 0.9 | G1 vs G3 | 6 | 0.652 | 0.143 | 3 |
| 0.9 | G2 vs G3 | 5 | 0.675 | 0.000 | 3 |

Abbreviations: G1, elite group; G2, second-level group; G3, ordinary group.
